# The phylogenetic structure of plant communities drives the belowground transmission of fungal pathogens

**DOI:** 10.1111/nph.71156

**Published:** 2026-04-06

**Authors:** Jose G. Maciá‐Vicente, Sofia I. F. Gomes, Eline A. Ampt, Justus Hennecke, Lisette M. Bakker, Jasper van Ruijven, Liesje Mommer

**Affiliations:** ^1^ Plant Ecology and Nature Conservation Wageningen University PO Box 47 6700 AA Wageningen the Netherlands; ^2^ Department of Microbial Ecology Netherlands Institute of Ecology (NIOO‐KNAW) PO Box 50 6700 AB Wageningen the Netherlands; ^3^ Marine Sciences and Applied Biology, Multidisciplinary Institute for Environmental Studies “Ramón Margalef” University Alicante PO Box 99 03080 Alicante Spain; ^4^ Institute of Biology Leiden University Leiden 2333 BE the Netherlands; ^5^ Forest Ecology and Forest Management Wageningen University PO Box 47 6700 AA Wageningen the Netherlands

**Keywords:** biodiversity, grasslands, pathogens, plant–soil feedback, roots, soil‐borne fungi

## Abstract

- Biodiversity is known to influence disease risk, yet the pathways of pathogen transmission within plant communities remain poorly understood, especially belowground. In particular, how soil‐borne pathogens move from resident vegetation and soil to colonize new hosts is unresolved.
- We traced belowground pathogen transmission using phytometer seedlings of two plant species planted in a long‐term grassland biodiversity experiment. After 3 months, we characterized the fungal communities of phytometer roots, resident plant roots, and soil using high‐throughput sequencing and the FungalTraits database to identify associations between pathogen taxonomy and plant families. Next, we related pathogen abundance to phytometer growth.
- The phylogenetic similarity of phytometers with resident plant species strongly predicted the relative abundance of pathogens that were considered family‐specific, but not of pathogens without a clear host preference. However, neither pathogen abundance in phytometers nor resident plant biomass affected phytometer growth, which was best explained by the resident communities' species richness.
- Combining sequencing of fungal communities with *in situ* field manipulations enabled us to track the associations between multiple soil‐borne pathogens and plant hosts within the full complexity of plant–soil systems. While pathogen dynamics were readily detectable, their consequences for plant performance may only become apparent over longer ecological timescales.

## Introduction

Plant pathogens are major drivers of natural plant community dynamics and ecosystem productivity (Paseka *et al*., 2020). They promote plant diversity and coexistence by controlling plant species that become too dominant, thereby enabling rarer ones to thrive (Bever *et al*., 2015; van Ruijven *et al*., 2020). Pathogens are thus hypothesized to reduce plant growth in species‐poor communities (i.e. monocultures; Maron *et al*., 2011; Bever *et al*., 2015; van Ruijven *et al*., 2020) compared to species‐rich communities. Host density is assumed to be higher in monocultures than in mixed communities, promoting the transmission and build‐up of specific pathogens that can impair growth and fitness (Freckleton & Lewis, 2006; Mordecai, 2011). However, if pathogen species have a more generalist lifestyle and can infect multiple plant species, even asymptomatically (Hersh *et al*., 2012; Malcolm *et al*., 2013), pathogen transmission may not be linearly correlated to host density (Power & Mitchell, 2004; Ampt *et al*., 2022a). This disconnect between host density and pathogen abundance – and eventually the development of disease symptoms – may be even more pronounced if pathogens can also multiply outside plant tissues (i.e. as soil saprotrophs; Termorshuizen & Jeger, 2008). Thus, the relationship between plant species richness, host density and pathogen abundance, and how their interactions affect plant growth remains unresolved, especially belowground.

The arguments of generalist lifestyles, that is, broad host ranges and ability to have ‘a life of their own’ are especially relevant for soil‐borne pathogenic fungi (Termorshuizen & Jeger, 2008). Soil‐borne pathogens often have broad host ranges (Newman & Derbyshire, 2020; Spear & Broders, 2021; Semchenko *et al*., 2022) and the ability to asymptomatically colonize the roots of numerous species (Malcolm *et al*., 2013; Kia *et al*., 2017; Lofgren *et al*., 2018). Moreover, a large variability in pathogenic performances within fungal species (Ma *et al*., 2010; Dong *et al*., 2015; Plissonneau *et al*., 2018) and the fact that generalist pathogens may still cause host‐specific effects through various mechanisms (Hersh *et al*., 2012; Benítez *et al*., 2013) make it challenging to define host specialization among local pathogen communities. The effects of pathogen communities have been frequently studied in plant–soil feedbacks (Petermann *et al*., 2008; Hendriks *et al*., 2013; Thakur *et al*., 2021), and high‐throughput amplicon sequencing approaches have allowed quantification of pathogen abundances (Wang *et al*., 2017; Ampt *et al*., 2022a; Maciá‐Vicente *et al*., 2023). However, elucidating the host specialization patterns among multiple soil‐borne pathogens in field experiments remains difficult, owing to the high complexity of potential interactions with plants (Barrett *et al*., 2009).

In this study, we take a next step by uncovering the belowground transmission pathways of specialist and generalist soil‐borne fungal pathogens in plant communities that differ in species composition. Previous studies have provided insights into the identity of soil‐borne pathogenic fungi in similar grassland communities (Leff *et al*., 2018; Ampt *et al*., 2022a; Maciá‐Vicente *et al*., 2023), but these studies have not correlated the pathogen abundance to plant growth, due to challenges in accounting for confounding factors and unbalanced designs (Vogel *et al*., 2019; Ampt *et al*., 2022a). Our experiment combined the study of ‘resident’ individuals in long‐term plant communities with semi‐controlled growth of seedlings of two plant species (phytometers) from the species pool. These phytometers served as pathogen traps, allowing us to monitor pathogen transmission from the other plant species in the community (resident plants) and the soil as well as to assess the impact of these pathogens on plant growth.

Because infection by pathogens – including soil‐borne fungal pathogens – generally is phylogenetically conserved among closely related plant species (Gilbert & Webb, 2007; Francioli *et al*., 2020), we used phylogenetic distance among resident species to infer host preferences among fungi, allowing us to distinguish between host‐associated and generalist pathogens. We hypothesized that: (1) the transmission of specialist pathogens from resident plants to phytometer seedlings decreases with the phylogenetic distance between them, indicating a higher pathogen pressure when related plant species co‐exist; and that (2) the increases in pathogen pressure in phytometer seedlings are negatively correlated to phytometer biomass, meaning that pathogen pressure translates into disease burden. Our analyses allowed us to trace the belowground pathways of pathogen transmission from residents and soil to seedlings, thereby identifying processes leading to the build‐up of pathogen communities and their subsequent effects on host growth.

## Materials and Methods

### Study site and experimental design

This study was done in a common garden experiment located at the experimental fields of Wageningen University & Research, the Netherlands. The experiment was established in April 2014 and consisted of 70 × 70 cm plots containing experimental plant communities varying in species richness and functional group composition (forbs and grasses), assembled from a pool of 16 perennial plant species typical of temperate grasslands. The experimental design encompasses a balanced representation of forb and grass species displaying diverse root traits (Bakker *et al*., 2018; see Supporting Information Methods S1 and Table S1 for details about the experiment establishment and treatments). In this study, we only used a subset of 120 plots containing 12 of the original 16 species, representing plant richness levels of 0 (*n* = 4), 1 (*n* = 44), and 4 (*n* = 72) species. The species excluded had almost disappeared from the experiment at the time of this study. Plant individuals that had been growing for several years in these plant communities are referred to throughout the text as *resident plants*.

To evaluate the impact of resident communities on the growth of newly established plants and the assembly of fungal communities in their roots, we used the species *Leucanthemum vulgare* (Asteraceae) and *Arrhenatherum elatius* (Poaceae) as phytometers. In May 2020, we planted 3‐wk‐old seedlings of each species (grown in trays with potting substrate in a glasshouse) in two opposite quadrants of each plot, using a 3 × 2 grid system with three seedlings per species 10 cm away from each other (Fig. S1). Phytometers that died within the first 2 wk were replaced.

### Collection of samples and measurements

In August 2020, 12 wk after planting the phytometers, we uprooted all phytometers and five individuals per resident species and plot to characterize their root‐associated fungal communities. We pooled the phytometer and resident samples by species per plot, separated the roots from shoots, and carefully washed the roots under running tap water. We then cut a *c*. 1 g subsample of pooled, clean roots per sample into 1–2 cm pieces that we stored at −80°C until DNA extraction. At this stage, we detected contamination in the seed batch used to plant the *A. elatius* phytometers with at least two other grass species. However, because the contaminant species were noticeably different from *A. elatius* only at the adult stage, we were able to discard them. This reduced the total number of individual *A. elatius* phytometers but did not affect the number of replicates, since most plots still contained at least one *A. elatius* phytometer (Methods S2).

Following the root sampling, we clipped all the remaining biomass from the resident plants in the plots to 2 cm above ground level and combined it by species with the aboveground biomass of residents collected previously. We determined the specific biomass of all the phytometer and resident species per plot after drying at 40°C.

Simultaneous with the root sampling, we collected composite bulk soil samples (*c*. 10 g) from each plot by combining soil cores taken near each plant sample using a 4 cm diameter auger. Each soil sample was split into two subsamples: one was immediately stored at −80°C until DNA extraction, whereas the remainder was stored at 70°C to measure pH, organic matter content (%), total C, and total N, and the contents of NH_4_^+^–N, NO_3_^−^–N, and PO_4_–P using standard procedures (Temminghoff, 2010).

### Fungal amplicon sequencing and analysis

We extracted total DNA from phytometer and resident root samples using the NucleoMag 96 Plant Kit (Macherey‐Nagel Gmgh & Co., Düren, Germany) on a KingFisher Flex Magnetic Particle Processor (ThermoFisher Scientific, Waltham, MA, USA), and from soil samples using the DNeasy Powersoil Kit (Qiagen, Venlo, the Netherlands). We profiled fungal communities following the procedures described in Maciá‐Vicente & Popa (2022), with a few modifications. In short, we amplified the ITS1 part of the fungal rDNA ITS region with primers ITS1F and ITS2 (White *et al*., 1990; Gardes & Bruns, 1993), and pair‐sequenced the amplicons on an Illumina MiSeq platform at Useq (Utrecht, the Netherlands). For sequencing, we randomly distributed samples from phytometer roots, resident roots, and soils in three DNA pools that were independently sequenced in three MiSeq runs. We assembled and quality‐filtered the sequence reads using the Dada2 pipeline (Callahan *et al*., 2017), followed by clustering of amplicon sequence variants into operational taxonomic units (OTUs) at 99% sequence similarity using Cd‐Hit v.4.6 (Fu *et al*., 2012). We taxonomically annotated the OTUs by comparison with the UNITE v.10.0 database (https://doi.org/10.15156/BIO/3301238; Kõljalg *et al*., 2005) and classified them into functional guilds by comparing the taxonomic annotations against the ‘primary lifestyle’ field of the FungalTraits database (Põlme *et al*., 2020).

### Data preparation and selection of predictor variables

We used R v.4.5.1 (R Core Team, 2025) for all data analyses. We discarded all samples with less than 1000 reads and all OTUs represented by less than five reads in the dataset, resulting in a final set of 497 fungal community samples: 202 from phytometer roots (101 of each *L. vulgare* and *A. elatius*), 175 from roots of different resident species, and 120 from soil (Table S1). The resulting dataset included 12 763 346 quality‐filtered reads representing 3848 fungal OTUs across phytometer roots (3020 039 reads, 1421 OTUs), resident roots (2710 702 reads, 1271 OTUs), and bulk soil (7032 605 reads, 3440 OTUs).

We incorporated three predictors in downstream analyses that summarize the phylogenetic relationships among plant species and communities in the experiment. The plant phylogeny used is based on that provided by Zanne *et al*. (2014), from which we extracted a subtree comprising only our focal species. The first predictor consisted of the first two phylogenetic eigenvectors obtained from a PCoA of the cophenetic distances between the tree tips. These represent the phylogenetic relationships between all individual species in our study, which we used as descriptors of host identity (‘ho’). The two eigenvectors only explained 15% of species‐level phylogenetic variation, but up to 85% at the family level, with the first eigenvector mostly separating Poaceae from eudicot families, and the second the Asteraceae from other families (Fig. S2). The second predictor was aimed at summarizing the relationships between phylogenetic structure between resident plant communities (‘ps’). We obtained it as the first two eigenvectors from a PCoA of mean pairwise phylogenetic distances between the plant species per plot, calculated with the *comdist()* function of package picante v.1.8.2 (Kembel *et al*., 2010). The last predictor consisted of a subset of ‘ps’, comprising the pairwise phylogenetic distances between each of the phytometer species and the resident communities (‘ppd’). We excluded the samples from plots with zero resident plant richness from all statistical analyses involving phylogenetic variables, as these could not be defined. For plotting purposes, we used the proportion of forb‐to‐grass resident species per plot, which closely represents the first eigenvector of the PCoA from community‐wide phylogenetic distances (Pearson's *ρ* = 0.99, *P* < 0.001), because it provides a more intuitive interpretation.

Additionally, we incorporated in the analyses potentially confounding factors that could affect results, such as sequencing runs, soil properties, or spatial factors. Only variables with significant effects were retained in each model, determined through automated variable selection or visual inspection of results. We calculated the spatial factors, summarizing the position of experimental plots, using Moran's eigenvector maps (Dray *et al*., 2006) using function *mem()* of package adespatial v.0.3‐21 (Dray *et al*., 2018). To meet normality assumptions in analyses, we used the log(*x*) transformation of soil PO_4_ content, and the log(*x* + 1) of soil NH_4_, NO_3_, and total N contents.

We did not account for the effects of the MiSeq sequencing run during the statistical analyses. A preliminary assessment showed that it explained 1.9% of total variation in fungal community structure (PERMANOVA, *F*_2_ = 5.5, *R* ^2^ = 0.019, *P* < 0.001), as compared to 12% explained by sample type (i.e. from residents, phytometers, or soil; *F*_2_ = 34.4, *R* ^2^ = 0.12, *P* < 0.001), and thus we considered its effect on downstream results negligible.

### Analyses of fungal community structure and composition

We analyzed the taxonomic composition of fungal communities using Krona charts (Ondov *et al*., 2011) and bar plots, and the patterns in community structure using PCoA ordinations after Hellinger transformation of OTU abundances (Legendre & Gallagher, 2001). To test the effects on fungal community structure of factors related to plant phylogeny, soil chemistry, and space, we used variation partitioning and PERMANOVA analyses as implemented in function *adonis2()* of package vegan v.2.6.6 (Oksanen *et al*., 2019). In both cases, only a subset of meaningful soil and spatial factors (Table S1) were included in each analysis, selected based on their explanatory power using function *best.r.sq()* of package mvabund v.4.2.1 (Wang *et al*., 2012). We also assessed collinearity among soil and spatial variables to ensure the independence of the selected variables within each set of factors. In the PERMANOVA analyses of data from resident roots, we restricted permutations within experimental plots using the *how()* and *Plots()* functions from the package permute v.0.9‐7 (Simpson *et al*., 2022), to account for the nested structure of residents' root samples within plots.

### Identification of plant family‐associated fungi

We identified OTUs with a significant association with the roots of resident plant species in the families Asteraceae or Poaceae (which include the two phytometer species) as an *ad hoc* proxy for a colonization preference toward a specific host family. While an arbitrary host taxon‐level cutoff likely underestimates the actual host ranges for many pathogens (Gilbert & Webb, 2007), the higher frequency of species from the Asteraceae and Poaceae in our experiment (Fig. S2) largely restricts OTU associations to the family level or to no specific family in the case of host generalists. Moreover, numerous studies show that both families assemble distinct root‐associated fungal communities, suggesting some degree of adaptation to either family (Mommer *et al*., 2018; Francioli *et al*., 2020; Maciá‐Vicente & Popa, 2022; Ampt *et al*., 2022a). Here, we explicitly favor the term ‘host‐associated’ to describe fungal preference for colonizing roots of phylogenetically related plant species, rather than ‘host‐specific’, which would have required plant bioassays to confirm disease induction on specific hosts.

We applied multispecies generalized linear models (GLMs) with the package mvabund v.4.2.1 (Wang *et al*., 2012) independently for each plant family, using a subset of the dataset including only resident plant communities and OTUs present in a minimum of 10 experimental plots. The method fits separate GLMs to individual OTU abundances while accounting for inter‐OTU correlation through resampling. To identify associations between OTUs and plant families, we included a binary variable encoding plant family membership as a predictor and we employed LASSO penalties (Brown *et al*., 2014) for model selection. OTUs with positive standardized coefficients were considered family‐associated, whereas those showing no correlation with either family were categorized as host generalists.

### Soil‐borne pathogens transmission and effects on phytometer biomass

We identified OTUs belonging to putative plant pathogens (henceforth plant pathogens) using the genus‐level guild annotations from FungalTraits. While this classification does not fully capture fungal ecological functions (Selosse *et al*., 2018; Põlme *et al*., 2020), it provides a practical classification for high‐throughput assessments of fungal diversity, including pathogens' occurrence patterns (Tanunchai *et al*., 2023; Lu *et al*., 2026). We further validated these assignments by searching the genus names against the USDA Fungus‐Host database, which compiles curated world‐wide records of fungal pathogens' occurrence (Farr *et al*., 2026). We used this to confirm whether frequently dominant genera are reported as pathogens across plant families (Table S2).

We used a model‐based fourth‐corner approach (Brown *et al*., 2014) using the *traitglm()* function in the mvabund package to create two models for each phytometer species. The method relies on species (OTUs) relative abundance data and species traits (host preference in this case, that is Asteraceae‐associated, Poaceae‐associated, or generalist OTUs) to estimate relationships between the predictor and trait variables. The first set of models included the mean phylogenetic distance between the phytometer and the resident community (‘ppd’) as a predictor, to test whether there is enhanced transmission of host‐associated pathogens between plant relatives (Hypothesis 1). The second set of models investigated relationships with phytometer biomass, to assess if the increased relative abundance of specific pathogens leads to impaired plant growth (Hypothesis 2). In both cases, we hypothesized negative relationships for pathogen specialists, reflecting lower root pathogen abundance in phytometers that: (1) grew alongside phylogenetically distant resident communities; and (2) exhibited less severe growth impairment. As in the previous models, we applied LASSO penalties to identify interaction coefficients that contribute significantly to results. Last, to create a baseline for the patterns observed in pathogens, we used the same model structures to test the relationships for all nonpathogenic OTUs.

### Relationships between phytometer biomass and plant community diversity

We investigated the relationship between plant richness and the aboveground biomass production of resident and phytometer species per plot using GLMs with Gamma or inverse Gaussian error distributions, after model selection and evaluating diagnostic plots. Then, we tested the relationship between the mean biomass per plot produced by each phytometer species and the mean phylogenetic distance between the phytometer species and the resident community (‘ppd’; Hypothesis 2) using GLMs with Gamma distribution of errors. In these models, we included spatial factors as covariates, selected from all soil and spatial variables based on their explanatory power via forward variable selection, using the *forward.sel()* function from package packfor v.0.08 (Dray *et al*., 2009).

### Structural equation models

We used piecewise structural equation modeling (SEM; Lefcheck, 2016) to identify the pathways of fungal pathogen transmission from resident communities to phytometer roots. We used it to partition the total effects of resident communities on phytometer growth into direct interactions (e.g. interspecific competition) and indirect effects mediated by the modulation of soil‐borne and root‐associated pathogen populations. This enabled us to distinguish between transmission occurring via soil vs direct root‐to‐root contacts, while simultaneously accounting for the relative contributions of resident community properties such as plant species richness and biomass. We built one independent model for each phytometer species using the *psem()* function from the piecewiseSem v.2.3.0 package (Lefcheck, 2016) and calculated direct, indirect, and total effects with confidence intervals based on bootstrapping with 10 000 replicates, using the *semEff()* function from package semeff v.0.6.1 (Murphy, 2022). The *a priori* models (Fig. S3) included the exogenous variable phytometer mean phylogenetic distance with residents (‘ppd’) as the main descriptor hypothetically driving the transmission of specific pathogens between related species (Hypothesis 1). We also included as exogenous variables the residents' richness and productivity per plot, as likely predictors of fungal occurrence and phytometer growth. Fungal variables represented the proportion of reads per sample for each of the host‐associated groups of fungal pathogens in soil and phytometer roots. Last, we tested whether pathogen build‐up impairs seedling growth (Hypothesis 2) by investigating the link between the relative abundance of host‐associated pathogens and phytometer biomass. We controlled for significant collinearity among variables by specifying them as correlated errors.

## Results

### Plant community structure shapes fungal communities in roots and soil

**Fig. 1:**
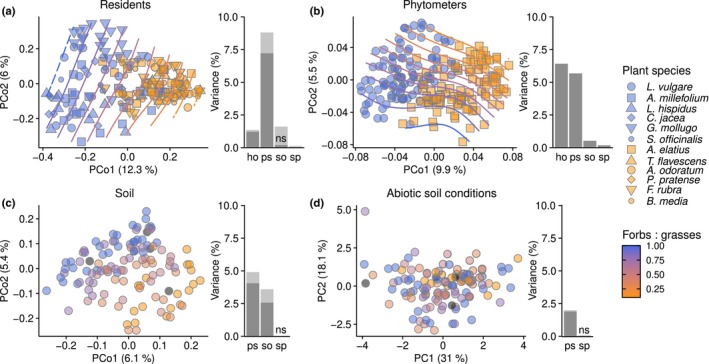
Variation of fungal communities and soil abiotic properties with plant communities. (a) A PCoA (left) shows the dissimilarities between fungal communities associated with roots of the resident plants. Point shapes differentiate plant species within forbs (blue) and grasses (yellow; see Supporting Information Fig. S2 for full species names); contour lines represent the proportion of forb‐to‐grass species in resident communities. The bar plot (right) represents the proportion of fungal community variation explained by the host species identity (ho), the phylogenetic structure of resident communities (ps), the abiotic soil conditions (so), and the spatial position of plots within the experiment (sp). Dark and light fractions of bars indicate exclusive variance explained by each factor and variance shared with other factors, respectively. (b) Variation of fungal communities associated with phytometer roots, as in (a). (c) Variation of fungal communities in bulk soil. Point colors are correlated with the forbs‐to‐grasses ratio in resident communities. Gray points correspond to plots with zero resident plant richness. The bar plot excludes the effect of host identity (ho), as it is not applicable. (d) PCA of soil chemical properties across experimental plots and variation partitioning analysis. The bar plot excludes the effects of host identity (ho) and soil properties (so). The values shown by all bars are significant at *P* ≤ 0.05, except where indicated (ns).

Illumina MiSeq ITS amplicon sequencing (Table S1) showed that fungal communities associated with roots of resident plants were primarily influenced by the phylogenetic structure of the plant community (‘ps’; 8.8% of total variance, based on variation partitioning analysis), particularly along the divide between forb‐to‐grass dominated plots (Fig. 1a). In comparison, the species identity of resident host species (‘ho’) had a much lower effect (1.4% of variance), and so did soil abiotic properties (‘so’) and spatial factors (‘sp’; Fig. 1a). Whereas the co‐occurrence of multiple resident species per plot could have some influence in these effects, PERMANOVA analyses accounting for interdependent measures confirmed the variation partitioning results (Table S3).

In contrast to residents, fungal communities in phytometer roots (Fig. 1b) were similarly affected by the phytometer species identity (‘ho’; 6.2% of variance) and the phylogenetic community structure (‘ps’; 5.7%). Moreover, these two spread along the first and second PCoA axes, respectively, indicating that they explain independent fractions of fungal community variation (Fig. 1b). Soil and spatial factors had small effects on fungal assembly in phytometer roots (< 1%; Fig. 1b). A separate analysis of the root fungal community of the two phytometer species confirmed the main effect of residents' community structure on the root‐associated fungi for each species, and the negligible effects by abiotic soil and spatial variables, particularly in *A. elatius* (Fig. S4).

The phylogenetic structure of resident communities also explained the largest amount of variation in bulk soil fungal communities (‘ps’; 4.9%), again determining a divide between forb‐ and grass‐dominated plots (Fig. 1c). In this case, soil parameters gained importance as predictors for fungal community assembly (‘so’; 3.6%), explaining a variance fraction largely independent of the one explained by resident communities (Fig. 1c). Contrastingly, plant community composition hardly influenced abiotic soil properties (2.3%; Fig. 1d). Spatial effects were negligible in explaining soil fungal communities and abiotic properties (Fig. 1c,d).

### Identification of host preferences in soil‐borne pathogens

Using the OTU annotations from FungalTraits and multispecies GLMs, we identified 20 OTUs of plant pathogenic fungi significantly associated with resident roots of Asteraceae species, 12 with Poaceae species, and 20 without a significant association with either family. Based on these associations, we classified the fungi as Asteraceae‐associated, Poaceae‐associated, and generalist plant pathogens (Table S4).

**Fig. 2:**
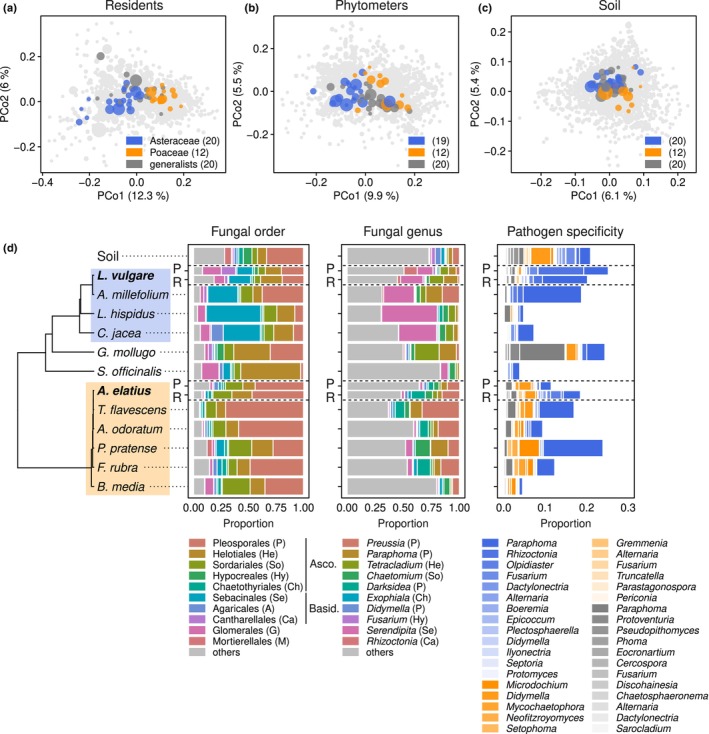
Identification of soil‐borne pathogens and their host preferences. (a) Operational taxonomic unit (OTU) scores of the PCoA showing fungal community variation in roots of resident plants (see Fig. 1a). Points correspond to individual OTUs, with point size correlated with their relative abundances. Points highlighted in blue, yellow, and dark gray correspond to OTUs significantly associated with Asteraceae, Poaceae, or generalist pathogens, respectively; light‐gray points correspond to all other OTUs. Numbers in the color key indicate the number of OTUs within categories. (b) OTU scores of the PCoA of phytometer roots (Fig. 1b). (c) OTU scores of the PCoA of bulk soil fungal communities (Fig. 1c). (d) Taxonomic composition of fungal communities across plant species and soil. The dendrogram represents the phylogenetic relationships among plant species, with Asteraceae and Poaceae species delimited by blue and yellow boxes, respectively (see Supporting Information Fig. S2 for full species names). The two bar plots leftward show the proportion of the 10 most abundant orders and genera across roots of plant species and in soil (pooled for all plots). Note that the bars for *Leucanthemum vulgare* and *Arrhenatherum elatius* are split for phytometers (P) and residents (R). The bar plot to the right shows the proportion of all the fungal OTUs identified as soil‐borne pathogens with a preference toward Asteraceae, Poaceae, or a generalist habit (blue, yellow, and dark gray, respectively).

The projection of individual OTUs onto the axes of the PCoA from fungal communities in resident roots confirmed their distribution along the first axis, encompassing the divide between forbs and grasses (Fig. 2a; compare to Fig. 1a). We observed an equivalent distribution of the OTUs along the forbs‐to‐grasses gradient when mapping them onto the ordinations for the phytometers (Fig. 2b) and bulk soil‐associated fungal communities (Fig. 2c). All the OTUs were represented in soil fungal communities (Fig. 2c), and all but one Asteraceae‐associated OTU among the phytometer‐associated fungi (Fig. 2b).

The taxonomic structure of fungal communities in roots and soil was consistent with other studies of fungal diversity in grassland soils and roots (Francioli *et al*., 2020; Ampt *et al*., 2022a), with a dominance of Ascomycota with the orders Pleosporales, Helotiales, Sordariales, and Hypocreales, followed by the Basidiomycota orders Sebacinales, Agaricales and Cantharellales (Figs 2d, S5). The OTUs classified as Asteraceae‐associated pathogens were distributed over 13 fungal genera, including some of the most abundant and widespread in the experiment, such as *Paraphoma*, *Rhizoctonia*, *Fusarium*, and *Didymella* (Fig. S2d). The Poaceae‐associated pathogens represented 11 genera, dominated by one OTU classified as *Microdochium* (Fig. 2d). Cross‐referencing these dominant genera against the USDA Fungus‐Host database confirmed their status of frequent plant pathogens, as compared to dominant, nonpathogenic genera in our study (Table S2). Although genus‐level records showed variable distributions across plant families, in cases they mirrored our results, most notably in the two most dominant genera, *Paraphoma* and *Microdochium*, with broad occurrences (> 30% of all records) in Asteraceae and Poaceae, respectively (Table S2).

Asteraceae‐associated OTUs showed greater relative abundance and distribution than Poaceae‐associated across samples, particularly those classified as *Paraphoma* (Fig. 2d). In this case, we validated that the relative proportion of dominant *Paraphoma*‐related OTUs was positively correlated with absolute abundances quantified by group‐specific qPCR (Methods S3; Fig. S6).

### Plant relatedness enhances the relative abundance of host‐associated pathogens without affecting growth

**Fig. 3:**
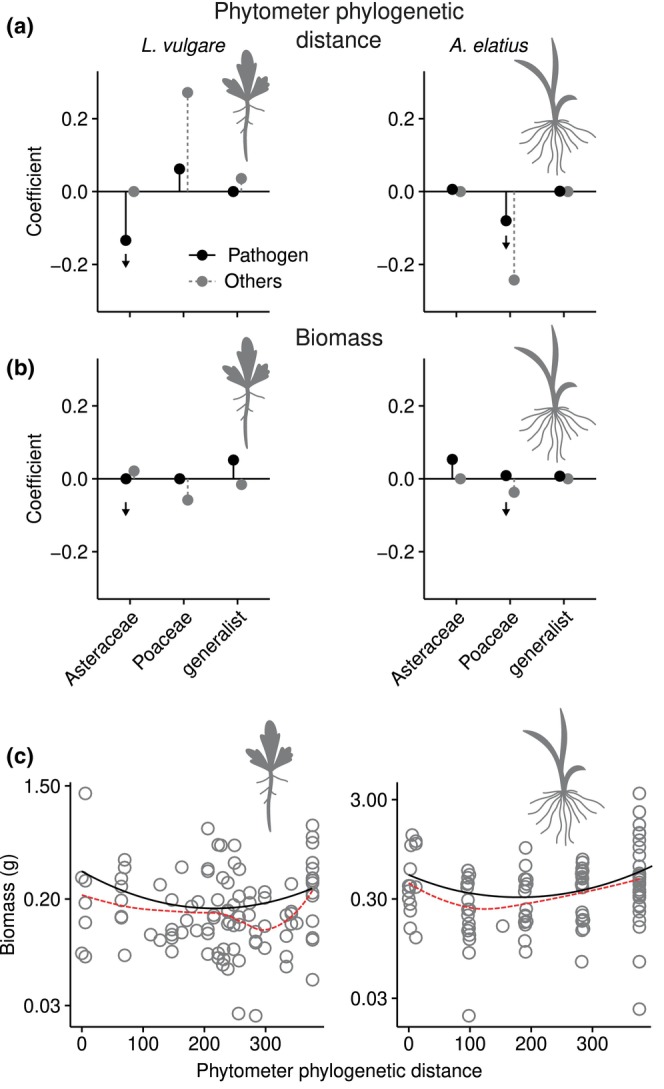
Transmission and effects on plant biomass of plant family‐associated soil‐borne pathogens. (a) Relationship between the abundance of host‐associated and generalist pathogens in each phytometer species (*Leucanthemum vulgare* and *Arrhenatherum elatius*) with the phylogenetic distance between phytometers and resident plant communities (‘ppd’). The coefficients indicate the direction and strength of the relationships, based on standardized estimates from a fourth‐corner model. Black points with solid lines show the values for soilborne fungal pathogens with a preference toward Asteraceae, Poaceae, or a generalist habit. For comparison, gray points with dashed lines represent the relationships for all nonpathogenic fungal OTUs. (b) Relationship between pathogen abundance in phytometer roots and aboveground biomass of phytometer plants, as in (a). The arrows in (a, b) highlight the direction of the relationships for host specialist pathogens hypothesized in this study. (c) Effect of phylogenetic distance between phytometers and the resident community (‘ppd’) on phytometers' growth. The black lines represent the regression estimates based on the best fitting generalized linear model, which had quadratic terms in both cases. The dashed red line represents the fitted values of loess regressions, highlighting the overall trend in the relationship between the variables. OTU, operational taxonomic unit.

The models to determine if the transmission of plant family‐associated pathogens is enhanced between closely related plant species (Hypothesis 1) showed negative correlation coefficients for both Asteraceae‐associated pathogens in *L. vulgare* and Poaceae‐associated pathogens in *A. elatius* (Fig. 3a), suggesting increased transmission of specific pathogens between plant relatives. However, the effect was weaker for *A. elatius*, which also showed an equivalent but stronger pattern for nonpathogenic Poaceae‐associated OTUs (Fig. 3a; gray, dashed line, and point), indicating that this effect is not exclusive for pathogens.

We applied a second set of fourth‐corner models to investigate if the accumulation of plant family‐associated pathogens in phytometer roots negatively impacts phytometer growth (Hypothesis 2), by regressing OTU abundance against plant biomass. In this case, the models indicated no negative relationship between relative pathogen abundance and phytometer growth, unlike our prediction (Fig. 3b). We also did not find linear relationships (*P* > 0.05) between phytometer growth and the mean phylogenetic distance between phytometers and resident communities (‘ppd’; Fig. 3c). The relationships for both phytometers were better explained by quadratic than linear models (*L. vulgare*: Akaike information criterion, AIC = −141.6 vs −138.7; *A. elatius*: AIC = 52.6 vs 56.5), suggesting that phytometers grow better when surrounded by either highly related or distant species (Fig. 3c). By contrast, resident species richness (Fig. S7) emerged as the primary, negative predictor of phytometer biomass for both *L. vulgare* (−0.89 ± 0.17 g per species, *F*_1,113_ = 13.1, *P* < 0.001) and *A. elatius* (−0.51 ± 0.17 g per species, *F*_1,109_ = 18.9, *P* < 0.001).

### Belowground transmission pathways of fungal pathogens

**Fig. 4:**
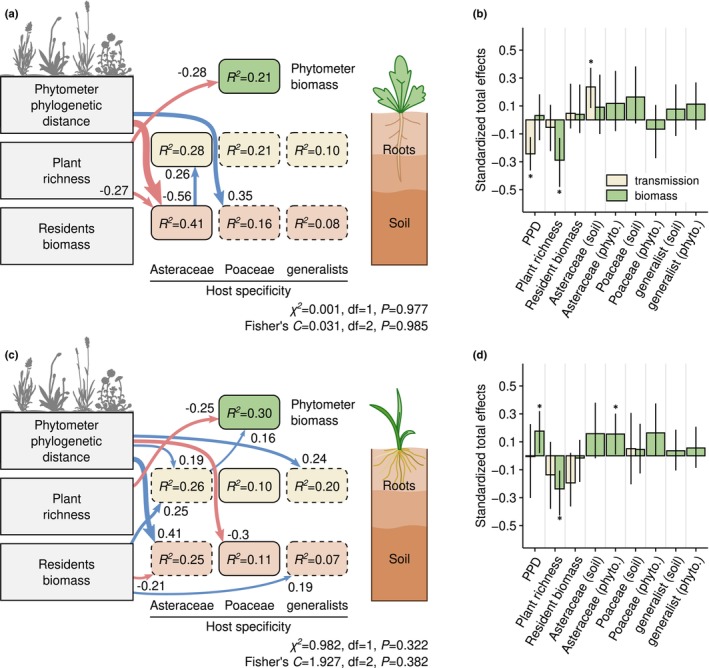
Belowground pathogen transmission and drivers of phytometer biomass. The diagrams show structural equation models (SEM) for the phytometer species *Leucanthemum vulgare* (a) and *Arrhenatherum elatius* (c). Boxes with sharp corners represent exogenous variables, and rounded boxes represent endogenous variables. The fungal variables consist of relative abundances in soil or phytometer roots, represented by boxes colored differently. Blue and red arrows represent significant positive and negative effects (*P* ≤ 0.05) between variables, respectively. The strength of the relationships is represented by the width of arrows and indicated by the adjacent values. In each diagram, the endogenous variables with solid borders indicate relationships included in this study's hypotheses. The bar plots (b, d) show the standardized total effects (sum of the direct and indirect effects) of variables explaining the transmission of host‐associated pathogens to phytometer roots (transmission), and the aboveground biomass of phytometers (biomass). The error bars delimit 95% confidence intervals based on 10 000 bootstrap replicates, and * indicates significant effects at *P* ≤ 0.05. PPD, phylogenetic distance between phytometers and resident plant communities.

We used SEM to further investigate the direct and indirect effects of resident community properties on the relationship between host‐associated pathogens' relative abundance and phytometer growth (Fig. 4; Table S5). These confirmed the negative relationship between mean phylogenetic distance (‘ppd’) and the accumulation of Asteraceae‐associated pathogens in *L. vulgare* phytometers. Phylogenetic distance was the main factor influencing the soil abundance of soil pathogens, which in turn positively affected their abundance in phytometer roots (Fig. 4a). When both direct and indirect effects are considered simultaneously, the abundance of Asteraceae‐associated pathogens in phytometers was equally explained by the negative relationship with phylogenetic distance to resident communities and a positive link to soil pathogen abundance (Fig. 4b), suggesting a belowground transmission pathway of specific pathogens from resident roots to soil, and from there to seedling roots. However, in line with previous models, the increase in relative abundance of Asteraceae‐associated pathogens in *L. vulgare* roots did not significantly influence phytometer growth, which instead was negatively related to plant richness (Figs 4a,b, S7).

Root colonization of *A. elatius* phytometers by Poaceae‐associated pathogens was unaffected by resident communities or the increase in relative abundance of such pathogens in soil, and neither were host‐associated pathogens related with the phytometer's biomass (Fig. 4c). As in *L. vulgare*, plant growth was primarily and negatively related to residents' species richness. In this case, we also found positive relationships with phylogenetic community distance (i.e. more biomass produced by phytometers when growing next to unrelated plant species), and root colonization by Asteraceae‐associated pathogens (Fig. 4d).

We detected in both phytometers an opposing transmission pattern between plant family‐associated pathogens and those with preferences towards the alternate phytometer species (i.e. positive in *L. vulgare* for Poaceae pathogens, and vice versa), further reflecting the host preferences of these fungal groups for their respective host lineages (Fig. 4a,c).

## Discussion

By integrating phylogenetic structure of plant communities, soil‐borne fungal communities, and phytometer growth, we studied how belowground transmission pathways shape pathogen accumulation in roots and, ultimately, influence host performance. Transmission of specialist pathogens from resident plants to phytometer seedlings decreased with increasing phylogenetic distance, reflecting a more efficient transmission among closely related hosts not observed in host generalist pathogens. However, this decreased pathogen proportion in phytometer roots in more distant communities did not translate to effects on phytometer biomass at the 3 months of the experiments' duration.

### Phylogenetic community structure determines the abundance of host‐associated pathogens

Our assessment of the plant communities' phylogenetic structure showed that plant family‐associated fungal pathogens showed a higher relative abundance in phytometer roots when phytometers grew near phylogenetically related resident species. Plant‐pathogenic fungi often affect multiple host species but exhibit preferences for species with shared evolutionary history, likely sharing morphological and physiological traits related to defense (Gilbert & Webb, 2007; Semchenko *et al*., 2022).

The analysis of overall fungal communities in the experiment revealed that the phylogenetic structure of plant communities has a significant impact on the root and soil mycobiota, with Asteraceae‐ and Poaceae‐associated communities diverging from one another and from those of other forbs (Fig. 1). During the establishment of the biodiversity experiment, all plots received the same amount of previously homogenized soil from a single source (Methods S1; Bakker *et al*., 2018), suggesting that they initially contained a uniform soil biota. Therefore, the variation of bulk soil fungal communities along the gradient of plant community composition at the sampling time reflects a selection of plant‐specific mycobiota since the experiment started. Additionally, the variation followed the primary axis separating forb‐ and grass‐dominated plots, which has been identified as a key predictor of soil‐borne and root‐associated fungal communities in both experimental and field settings (Heinen *et al*., 2020; Maciá‐Vicente & Popa, 2022; Ampt *et al*., 2022a). Interestingly, the legacy of plant communities on soil biota was not mirrored by soil abiotic conditions, suggesting that the selective effect on the soil biota is directly driven by plant growth rather than indirectly by changes in the abiotic soil environment. The selection of soil biota specific for plant communities sets the stage for plant–soil feedbacks, including the efficient transmission of natural enemies between susceptible host species (Eisenhauer *et al*., 2012; van Ruijven *et al*., 2020).

Our results support our first hypothesis of a link between host‐associated pathogens' transmission and host relatedness. This implies that communities with many phylogenetically related plant species provide higher densities of susceptible hosts for certain soil‐borne pathogens, increasing the likelihood of infection and build‐up within host tissues (Burdon & Chilvers, 1982; McCallum *et al*., 2001). Thus, our study suggests a link between host density and transmission of fungal pathogens with host preferences (rather than disease expression) in the field. Host specialization and density dependence are central tenets of epidemiology (Bell *et al*., 2006; Freckleton & Lewis, 2006; Mordecai, 2011) whose testing in the field has mainly relied on measures of disease development (e.g. incidence or severity of symptoms; Packer & Clay, 2000; Bell *et al*., 2006; Miller *et al*., 2019) rather than tracking pathogen populations. Previous studies have reported population changes of soil‐borne pathogens consistent with pathogen dilution and spillover in the field (Heinen *et al*., 2020; Ampt *et al*., 2022a; Maciá‐Vicente *et al*., 2023; Wang *et al*., 2023), but explicit tests have been mainly conducted under heavily controlled settings (Benítez *et al*., 2013; Ampt *et al*., 2022b).

### Belowground transmission of host‐associated pathogens varies with host lineage

The transmission of specialist pathogens was particularly evident for Asteraceae‐associated pathogens in *L. vulgare* phytometers. This aligns with previous studies based on the same biodiversity experiment, where Asteraceae‐associated *Paraphoma* spp., the dominant soil‐borne pathogen in our study, showed similar distribution patterns (Francioli *et al*., 2020; Ampt *et al*., 2022a) and disease development in potted *L. vulgare* seedlings (Mommer *et al*., 2018). However, results were less clear for Poaceae‐associated pathogens in *A. elatius*, whose relationship with the presence of neighboring grasses was weaker than in Asteraceae (Fig. 3a). While we confirmed a correlation between sequence‐based relative abundances and absolute abundance in *Paraphoma* spp. (Fig. S6), suggesting a genuine build‐up of this pathogen in *L. vulgare* roots, we cannot assume this relationship holds for other taxa. This includes the dominant, strongly grass‐associated *Microdochium*, for which we did not perform absolute quantification.

The differing results between the two phytometer species may also indicate variations in host quality (Mommer *et al*., 2025) between Asteraceae and Poaceae – including differences in root traits and susceptibility to infection, or likelihood of transmitting pathogens. The differences between families can also reflect the experiment's local conditions, since pathogen performance likely is influenced by the unique pathogen species pool and local environmental and historical factors (Hersh *et al*., 2012; Lin *et al*., 2012; Swinfield *et al*., 2012). Soils in our experiment had greater frequency and abundance of Asteraceae‐associated than Poaceae‐associated pathogens (Fig. 2), a trend consistent with previous studies on this site (Francioli *et al*., 2020; Ampt *et al*., 2022a) that could have facilitated detecting changes in the Asteraceae‐associated pathogens. Alternatively, the difference between families may stem from a lower sensitivity of the phylogenetic distance metric in grasses, owing to the smaller distances among grass species as compared to forbs (Fig. S2).

Overall, our findings highlight that diverse plant–soil interactions may simultaneously affect distinct plant hosts coexisting in a community. However, these results may also reflect the insufficient resolution in the identification of many fungi – a drawback intrinsic to high‐throughput sequencing studies (Nilsson *et al*., 2018) – or lack of knowledge on their ecologies. For instance, the second‐most abundant OTU in Poaceae roots (OTU3051), both of residents (13% relative abundance in grasses vs 2.7% in Asteraceae) and *A. elatius* phytometers (9%), displayed a strong host preference towards the family (Table S4). Despite its high ITS sequence similarity with numerous uncultured and unidentified records in NCBI GenBank, we could only identify this OTU at the order level in the Pleosporales and thus did not assign it to a functional guild. Because this and 15 other unidentified OTUs (compared to six among Asteraceae‐associated fungi) strongly influenced the association between non‐pathogenic fungi in *A. elatius* phytometers and the proportion of resident grasses (Fig. 3a; gray dashed line and point), our results might have differed if these OTUs were better taxonomically and ecologically identified. This showcases the need for research on the organismal biology of soil fungi to understand their effects on plant growth.

### The increase in pathogens' relative abundance does not affect phytometers' growth

Contrary to our second hypothesis, the accumulation of plant family‐associated pathogens in either phytometer did not affect the growth of the seedlings, nor did total plot biomass. Instead, phytometer biomass was directly related to the species richness of the resident communities, suggesting that plant species competition, more than pathogens' effects, may influence the growth of newly established seedlings. This finding aligns with previous studies indicating that negative plant–soil feedbacks may not be as important in determining biomass in the field as expected (Fitzpatrick *et al*., 2017), or that it may require longer time‐scales than covered by our study (Cardinale *et al*., 2007; in 't Zandt *et al*., 2021).

Pathogens may impact plant community dynamics more by affecting seedling survival than by influencing individual plant biomass (Van der Putten & Peters, 1997; Lin *et al*., 2012; Swinfield *et al*., 2012). Seed and seedling pathogens, such as those causing damping‐off, cause high mortality among plant offspring (Jarosz & Davelos, 1995). As hosts mature, pathogen effects may become less obvious (Ampt *et al*., 2022b), require higher pathogen abundance to inflict damage (Thrall *et al*., 1997), or become reduced when other microbial endophytes colonize the host (Hersh *et al*., 2012; Durán *et al*., 2018). Overall, the impact of pathogen pressure on the growth of small seedlings may add little to the bulk of community biomass, whereas changes in seedling survival are more likely to substantially affect total biomass by determining the extent of adult plant contributions at later stages. Further studies should thus investigate variables beyond plant biomass, such as plant survival or fitness, and evaluate long‐term effects on plant‐community structure rather than focusing solely on the growth of individual plants.

Additionally, the differential recruitment of beneficial microbiota by plant species may affect pathogen infection, disease progression, or mitigate negative impacts on growth via growth‐promoting effects (Wehner *et al*., 2010). In this study, mutualistic root‐colonizing fungi were strongly represented in Asteraceae plant roots, such as Glomeromycota (forming arbuscular mycorrhizas) in *L. vulgare* phytometers (18% relative abundance) and Sebacinales (14–50% vs 1–8% in other plant species; Fig. S5). These groups are hypothesized to alleviate pathogen pressure (Weiß *et al*., 2016; Eck *et al*., 2022), indicating that distinct associations with soil microbiota among plant lineages may influence outcomes. Investigating how soil biota affect pathogen dynamics and function in ecosystems is essential to understand how disease pressure shapes plant communities. However, addressing this will require dedicated, manipulative field studies capable of managing the complex interplay of factors and interactions present in natural environments, which was outside the scope of our study.

### Conclusions

We showed that plant community phylogenetic structure predicts the belowground abundance of host‐associated pathogens, with stronger effects in Asteraceae than in Poaceae, and that these pathogens transmit mainly via soil rather than by alternative pathways such as direct root contacts. Surprisingly, however, neither pathogen abundance nor resident biomass explained seedling performance, which was instead best predicted by plant species richness. This highlights a decoupling between pathogen transmission and short‐term plant growth, suggesting that the ecological consequences of belowground pathogen dynamics may only manifest over longer timescales.

## Competing interests

None declared.

## Author contributions

JGM‐V, SIFG, EAA, LMB, JR and LM designed the research. JGM‐V, SIFG, EAA and JvR conducted fieldwork and performed experiments. JGM‐V, SIFG, EAA and JH analyzed the data. JGM‐V drafted the manuscript. All authors contributed to the interpretation of the results and the writing of the final version of the manuscript.

## Disclaimer

The New Phytologist Foundation remains neutral with regard to jurisdictional claims in maps and in any institutional affiliations.

## Supporting information


**Fig. S1** Layout of experimental grassland plots with planted phytometers.
**Fig. S2** Phylogenetic relationships between the plant species in this study and inclusion of phylogenetic distances in statistical analyses.
**Fig. S3** *A priori* structural equation model.
**Fig. S4** Variation of fungal communities in roots of the two phytometer species, *Leucanthemum vulgare* and *Arrhenatherum elatius*.

**Fig. S5** Interactive Krona charts summarizing the taxonomic composition of fungal communities.
**Fig. S6** Comparison of the relative abundance of *Paraphoma* sp. OTUs with absolute quantification via qPCR.
**Fig. S7** Relationship of residents and phytometers biomass with plant richness.
**Methods S1** Description of the long‐term biodiversity experiment.
**Methods S2** Contamination of *Arrhenatherum elatius* phytometers with different grass species.
**Methods S3** Comparison of qPCR and amplicon sequencing estimates of fungal abundance.

**Table S1** Details of the experimental grassland plots included in this study.
**Table S2** Summary of dominant fungal genera cross‐referenced with the USDA Fungus‐Host database.
**Table S3** PERMANOVA results showing the effects of plant phylogeny, community diversity, soil chemistry, and spatial factors on root and soil fungal communities.
**Table S4** List of host‐specific plant pathogenic OTUs.
**Table S5** SEM scores.
Please note: Wiley is not responsible for the content or functionality of any Supporting Information supplied by the authors. Any queries (other than missing material) should be directed to the *New Phytologist* Central Office.

## Data Availability

The Illumina MiSeq sequence data generated is available at the NCBI Sequence Read Archive under BioProject number PRJNA1294973. The datasets are available online at Figshare (doi: 10.6084/m9.figshare.29579627).
