# Supplementary material for: The phylogenetic structure of plant communities drives the belowground transmission of fungal pathogens
Source: New Phytol. 2026 Apr 6;250(6):3976–89. doi: 10.1111/nph.71156 (PMC13193493; doi:10.1111/nph.71156)
Supplement: Supplementary file 1 — Fig. S1 Layout of experimental grassland plots with planted phytometers. Fig. S2 Phylogenetic relationships between the plant species in this study and inclusion of phylogenetic distances in statistical analyses. Fig. S3 A priori structural equation model. Fig. S4 Variation of fungal communities in roots of the two phytometer species, Leucanthemum vulgare and Arrhenatherum elatius. [file NPH-250-3976-s002.pdf]

New Phytologist **Supporting Information**

Article title: **The phylogenetic structure of plant communities drives the belowground transmission of fungal pathogens**

Authors: Jose G. Maciá-Vicente, Sofia I.F. Gomes, Eline A. Ampt, Justus Hennecke, Lisette M. Bakker, Jasper van Ruijven, Liesje Mommer

Article acceptance date: 17 March 2026

The following Supporting Information is available for this article:

Fig. S1 Layout of experimental grassland plots with planted phytometers.

Fig. S2 Phylogenetic relationships between the plant species in this study and inclusion of phylogenetic distances in statistical analyses.

Fig. S3 *A priori* structural equation model.

Fig. S4 Variation of fungal communities in roots of the two phytometer species, *Leucanthemum vulgare* and *Arrhenatherum elatius*.

Fig. S5 Interactive Krona charts summarizing the taxonomic composition of fungal communities.

Fig. S6 Comparison of the relative abundance of *Paraphoma* sp. OTUs with absolute quantification via qPCR.

Fig. S7 Relationship of residents and phytometers biomass with plant richness.

Table S1 Details of the experimental grassland plots included in this study.

Table S2 Summary of dominant fungal genera cross-referenced with the USDA Fungus-Host database.

Table S3 PERMANOVA results showing the effects of plant phylogeny, community diversity, soil chemistry, and spatial factors on root and soil fungal communities.

Table S4 List of host-specific plant pathogenic OTUs.

Table S5 SEM scores.

Methods S1 Description of the long-term biodiversity experiment.

Methods S2 Contamination of *Arrhenatherum elatius* phytometers with different grass species.

Methods S3 Comparison of qPCR and amplicon sequencing estimates of fungal abundance.

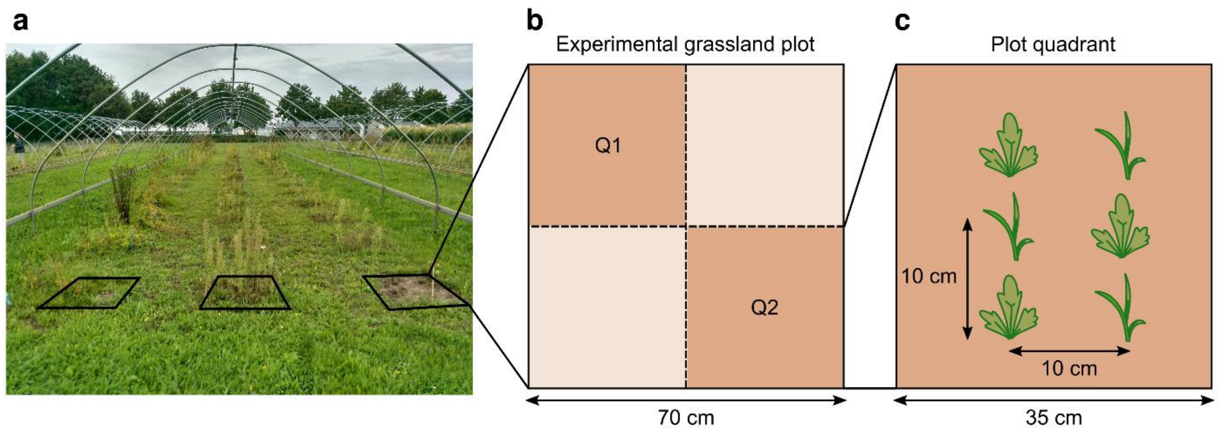

Fig. S1 Layout of experimental grassland plots with planted phytometers. (a) Photograph of the biodiversity experiment highlighting three experimental plots at the forefront. (b) Diagram of the division of plots into four quadrants, two of which (Q1 and Q2) contained the phytometer plants. (c) Diagram of one quadrant displaying the position and distances between phytometer plants. Each quadrant included three *Leucanthemum vulgare* and three *Arrhenatherum elatius* phytometers (represented by distinct plant drawings.)

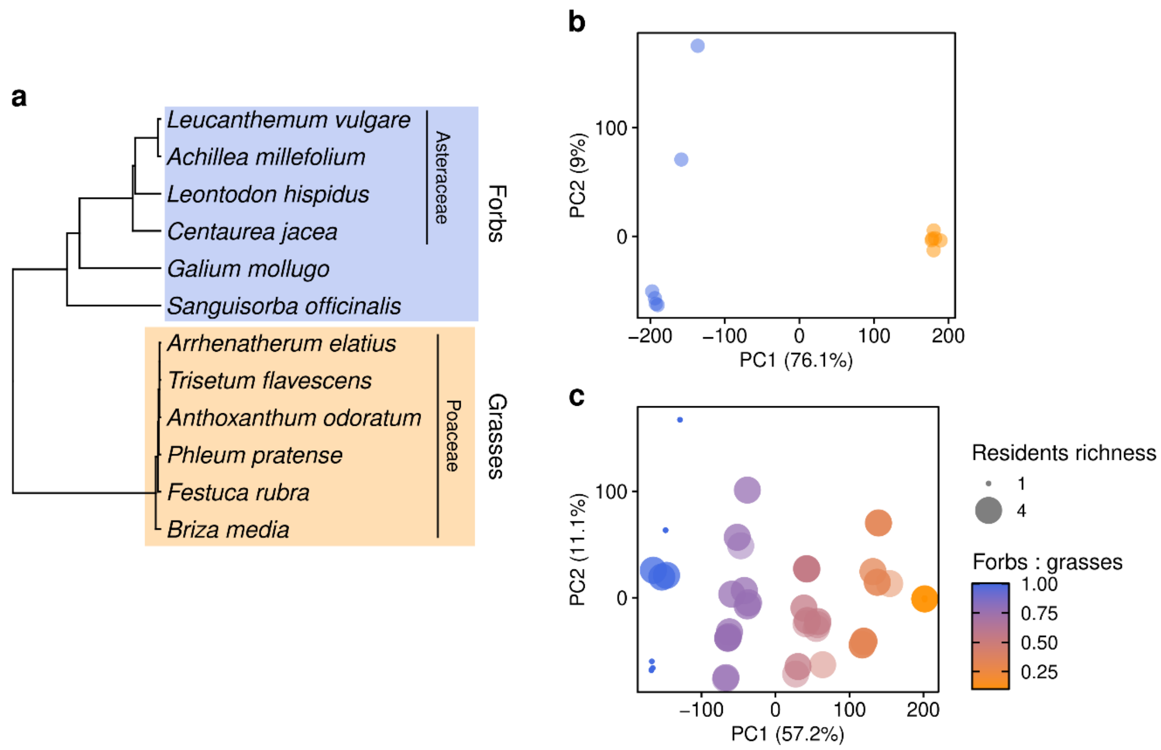

Fig. S2 Phylogenetic relationships between the plant species in this study and inclusion of phylogenetic distances in statistical analyses. (a) Phylogenetic tree displaying the evolutionary relationships between the plant species. (b) Principal coordinates analysis (PCoA) ordination the cophenetic distances shown in the tree in a. Each point corresponds to a plant species. The position of points is slightly jittered to avoid overlaps. (c) PCoA displaying the mean phylogenetic community distances across experimental plots. Each point corresponds to an experimental grassland plot. Point colors are correlated with the forbs-to-grasses ratio in resident communities, and point sizes indicate species richness.

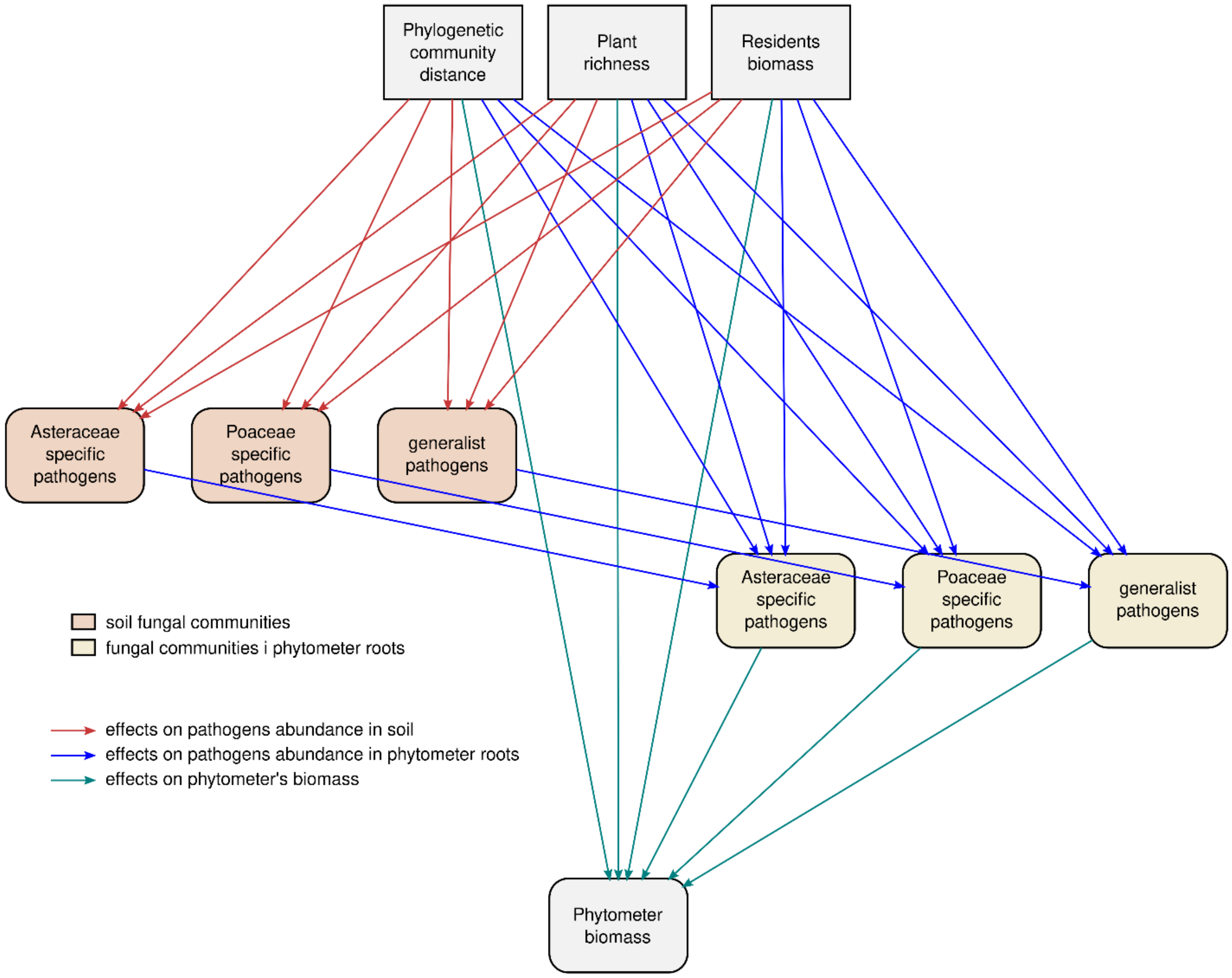

Fig. S3 *A priori* structural equation model (SEM) testing the direct and indirect effects from plant community properties and pathogen abundance on phytometers' biomass.

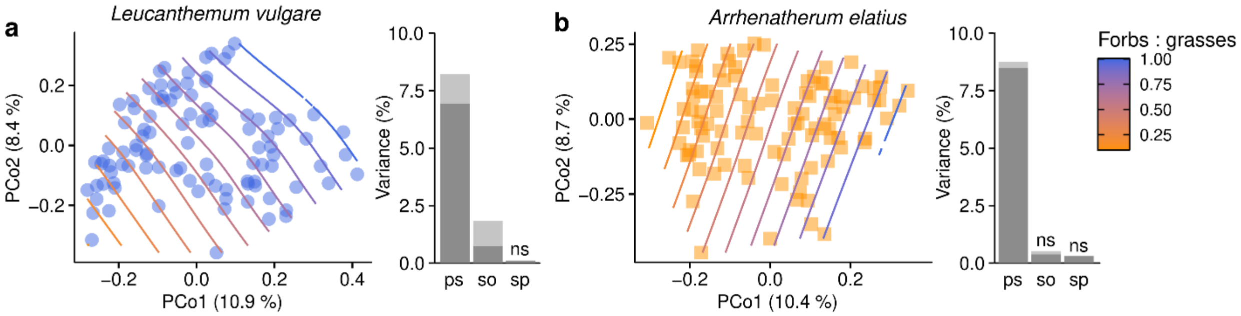

Fig. S4 Variation of fungal communities in roots of the two phytometer species, *Leucanthemum vulgare* (a) and *Arrhenatherum elatius* (b). Plots in the left of each panel show principal coordinates analysis (PCoA) representing the dissimilarities between fungal communities associated with roots of each phytometer species. Contour lines represent the ratio of forbs-to-grasses in resident communities. The bar plot (right) represents the proportion of fungal community variation explained by the phylogenetic structure of resident communities (ps), the abiotic soil conditions (so), and the spatial position of plots within the experiment (sp). Dark and light fractions of bars indicate exclusive variance explained by each factor and variance shared with other factors, respectively. The values shown by all bars are significant at $P \leqslant 0.05$, except where indicated (ns).

(Provided in a separate file)

Fig. S5 Interactive Krona charts summarizing the taxonomic composition of fungal communities in phytometer roots, residents' roots, and bulk soil.

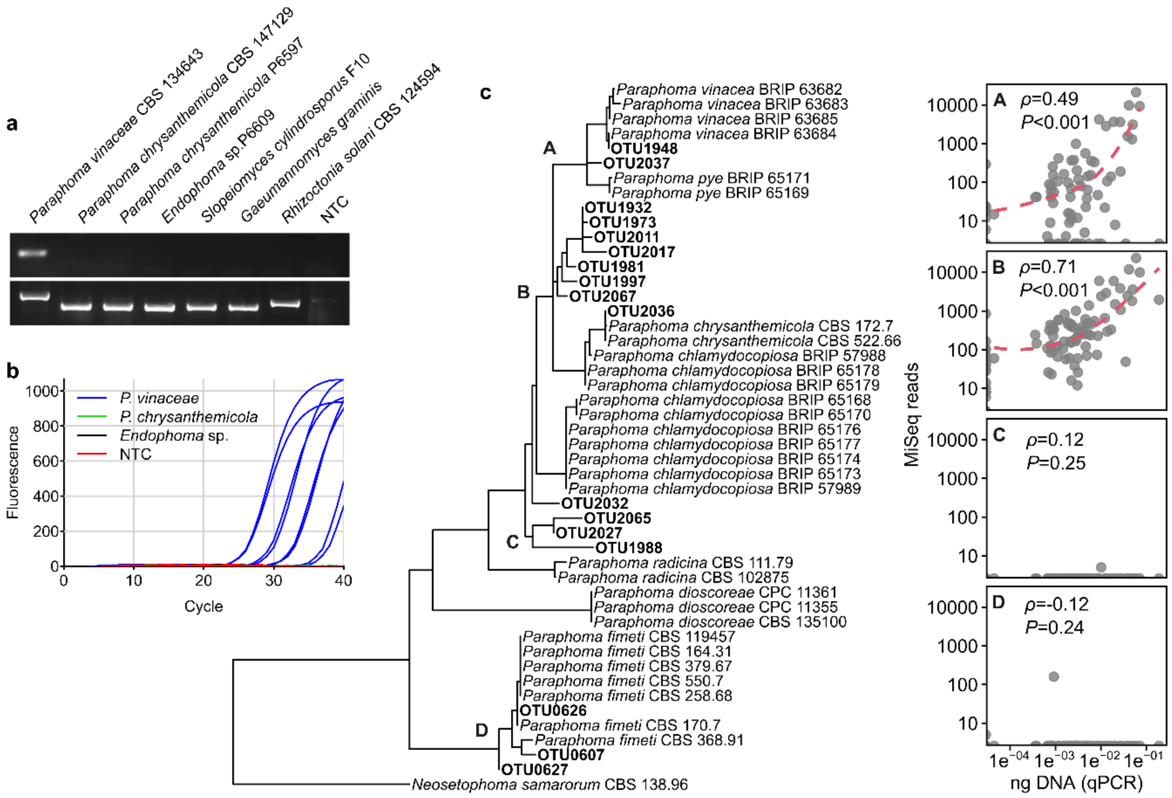

Fig. S6 Comparison of the relative abundance of *Paraphoma vinacea*-related operational taxonomic units (OTUs) with absolute quantification via group-specific quantitative PCR. (a) PCR amplification of *P. vinaceae* DNA with specific primers designed for the species, showing a lack of amplification in other species (top lane). As positive control, all DNA extracts were amplified with general primers (ITS1F/4) targeting the fungal ITS region (bottom lane). NTC stands for 'non-template control'. (b) Quantitative PCR (qPCR) assay to assess the species-specificity of the method, using serial dilutions of genomic DNA extracted from a *P. vinacea* culture, and from other two related fungal species. Note amplification only for *P. vinacea*, indicating that the assay is species-specific. (c) Maximum-likelihood phylogenetic tree based in multilocus sequencing (ITS, LSU, and *tef-1α*) including representatives of all species in genus *Paraphoma*, and all OTUs in this study classified in the genus. Capital letters indicate different OTU selections used to correlate their cumulative abundances with qPCR-based quantification. The scatterplots to the right show the correlation between Illumina-based relative abundances, and qPCR-based absolute abundances in each case. The plots C and D with OTUs clustering away

from *P. vinacea* are shown for reference. Values within plots indicate Spearman’s correlation (ρ) between variables, with p-values.

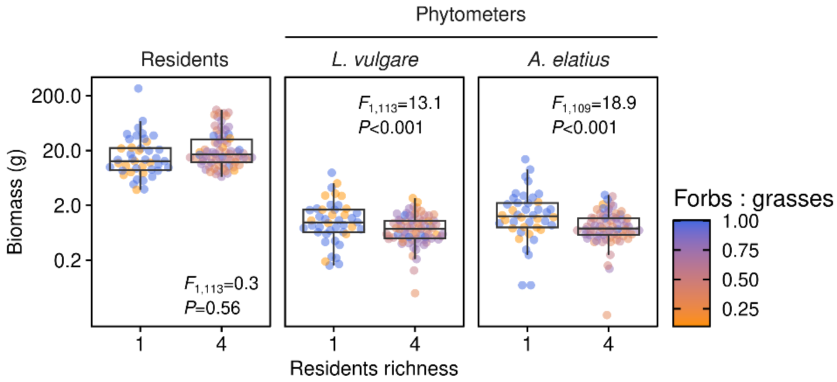

Fig. S7 Relationship of residents and phytometers biomass with plant richness. The boxplots show the total aboveground dry biomass of the resident species and the phytometers *Leucanthemum vulgare* and *Arrhenatherum elatius* in plots with 1 or 4 plant species. Individual points represent biomass values for each plot. Point colors are correlated with the forbs-to-grasses ratio in resident communities.

Table S1 Details of the experimental grassland plots included in this study. **(Provided in a separate file)**

Table S2 Summary of dominant fungal genera cross-referenced with the USDA Fungus-Host database. Values indicate the total number and percentage (in parentheses) of records identified as pathogens, including specific occurrences across relevant plant families. Genera where a high association (>30%; arbitrary threshold) with either Asteraceae or Poaceae plant families are highlighted in bold-face text. The raw data for this analysis are available online at Figshare (https://doi.org/10.6084/m9.figshare.29579627). **(Provided in a separate file)**

Table S3 PERMANOVA results showing the effects of plant phylogeny, community diversity, soil chemistry, and spatial factors on root and soil fungal communities.

		Phytometer roots			Residents' roots			Soil		
		R^2	*F_1*	*P*	*R^2*	*F_1*	*P*	*R^2*	*F_1*	*P*
Plant communities [1]	Phylogenetic eigenvector 1 ('ho')	**0.066**	**15.2**	**0.001**	**0.009**	**1.7**	**0.011**	n.d.	n.d.	n.d.
	Phylogenetic eigenvector 2 ('ho')	n.d.	n.d.	n.d.	**0.014**	**2.7**	**0.001**	n.d.	n.d.	n.d.
	Phylogenetic community eigenvector 1[b] ('ps')	**0.037**	**8.5**	**0.001**	**0.058**	**11.3**	**0.001**	**0.034**	**4.4**	**0.001**
	Phylogenetic community eigenvector 2 ('ps')	**0.02**	**4.6**	**0.001**	**0.017**	**3.4**	**0.001**	**0.017**	**2.1**	**0.001**
	Plant richness	**0.006**	**1.5**	**0.027**	**0.008**	**1.5**	**0.042**	**0.011**	**1.5**	**0.009**
Soil chemistry[2]	Nitrogen	n.d.	n.d.	n.d.	**0.007**	**1.4**	**0.047**	**0.021**	**2.7**	**0.001**
	NO_3	n.d.	n.d.	n.d.	n.d.	n.d.	n.d.	**0.021**	**2.7**	**0.001**
	Organic matter	0.005	1.3	0.117	n.d.	n.d.	n.d.	n.d.	n.d.	n.d.
	pH	**0.007**	**1.5**	**0.021**	n.d.	n.d.	n.d.	**0.015**	**1.9**	**0.001**
	PO_4	0.005	1.2	0.193	0.005	1.1	0.318	**0.011**	**1.5**	**0.008**
Spatial factors[3]	MEM101	**0.007**	**1.5**	**0.005**	n.d.	n.d.	n.d.	n.d.	n.d.	n.d.
	MEM120	n.d.	n.d.	n.d.	0.004	0.8	0.913	n.d.	n.d.	n.d.

MEM210	n.d.	n.d.	n.d.	n.d.	n.d.	n.d.	0.008	1	0.465
MEM221	n.d.	n.d.	n.d.	n.d.	n.d.	n.d.	0.009	1.1	0.144
MEM249	0.004	1	0.563	n.d.	n.d.	n.d.	n.d.	n.d.	n.d.
MEM267	n.d.	n.d.	n.d.	n.d.	n.d.	n.d.	0.007	0.9	0.692
MEM328	n.d.	n.d.	n.d.	0.007	1.3	0.085	n.d.	n.d.	n.d.
MEM366	n.d.	n.d.	n.d.	0.005	1	0.400	n.d.	n.d.	n.d.
MEM443	0.004	0.9	0.649	n.d.	n.d.	n.d.	n.d.	n.d.	n.d.

PERMANOVA analysis of fungal communities in phytometer roots, residents' roots, and soil used different sets of soil chemistry and spatial factors, based on a previous forward selection of variables likely to be associated with the data. Hence the cells with n.d. (not determined) in these variable categories. [1]Phylogenetic eigenvectors summarize the phylogenetic placement of host plants for root-associated fungal communities. Eigenvector 1 represents the division between Poaceae and eudicots, and eigenvector 2 between Asteraceae and other families (Fig. S2). Phylogenetic community eigenvectors summarize the phylogenetic composition of resident communities in a similar way to phylogenetic eigenvectors. [2]Non-normally distributed soil variables were transformed before using in statistical tests: NO_3 content was *log(x + 1)* transformed, and PO_4 content log(x)-transformed. [3]Spatial factors are represented by Moran's eigenvector maps (MEMs).

Table S4 List of host-specific plant pathogenic OTUs. **(Provided in a separate file)**

Table S5 Structural equation model (SEM) scores. **(Provided in a separate file)**

Methods S1 *Description of the long-term biodiversity experiment*. The long-term biodiversity experiment where this study was done is located at the experimental fields of Wageningen University & Research, The Netherlands (51.99 N, 5.66 E), and has been described in detail elsewhere (Bakker *et al.*, 2018; Francioli *et al.*, 2020a; Ampt *et al.*, 2022). The experimental design emphasized a balanced representation of forb and grass species, displaying a diversity of root traits independent of plant phylogeny and using species characteristic of European temperate grasslands (Bakker *et al.*, 2018). The main exception in the representation of wild species is the absence of legumes in the experiment, which were actively avoided during the set-up due to the disproportionate effect they usually have on community productivity, which would mask specific biomass changes in other species (van Ruijven & Berendse, 2003). The forb and grass species were grown in monocultures and species mixtures differing in composition to assess the importance of species richness and root trait diversity for productivity.

The experiment was established in April 2014 in a field where soil had been replaced with a lower layer of pure river sand (50-80 cm depth) and an upper layer (0-50 cm) of a 3:1 mixture of river sand with soil from an old field. The experiment consisted of 198 70×70 cm plots, delimited by 22 cm-deep wooden frames open at bottom, containing experimental plant communities varying in species richness (0, 1, 4, or 16 species) and functional group composition (forbs and grasses). The communities were assembled from a pool of 16 perennial plant species typical of temperate grasslands, originally planted at a rate of 64 similarly sized seedlings per plot in a 8×8 cm grid (Bakker *et al.*, 2018), but that showed variable specific coverages at the time of this study (2020). The diversity gradient in the experiment was maintained by periodically weeding off spontaneous species different from those planted.

In this study we only used plots with plant richness of 0 (n = 4), 1 (n = 44), and 4 (n = 72) species. Whereas the original experiment comprised 16 grassland species (Bakker *et al.*, 2018), we focused on 12 of these species, because the other four (*Agrostis stolonifera*, *Festuca pratensis*, *Prunella vulgaris*, and *Ranunculus sardous*) species had almost disappeared from the experiment after several years, a pattern commonly found long-term biodiversity experiments (Weisser *et al.*, 2017). We excluded all plots from the main experiment comprising these species (both monocultures and mixtures).

Methods S2 *Contamination of* Arrhenatherum elatius *phytometers with different grass species*. During the samples collection, we detected a contamination in the seed batch used to plant the *A. elatius* phytometers with at least two other grass species. This contamination went unnoticed during the steps of seed germination and planting in the field, due to the absence of morphological differences between grass seedlings. However, the contaminant species were noticeably different from *A. elatius* at the adult stage, when we were able to sample them separately. Although we processed these samples following the same steps of biomass measurements and characterization of root-associated fungal communities, we did not include such data in subsequent analyses.

The seed batch contamination resulted in a considerable difference in the number of individual phytometers recovered from each species upon sampling collection, with a total of 498 (mean ± SD, 4.9 ± 1.4; median, 5 per plot) *Leucanthemum vulgare* phytometer individuals, and 312 (mean ± SD 2.9 ± 1.7; median 3) *A. elatius* individuals. Nevertheless, the contamination did not affect the number of plot replicates, since at least one living *A. elatius* phytometer individual was present in as many plots as there were living *L. vulgare* phytometers (101 plots in either case).

For *L. vulgare*, we calculated the survival rate by dividing the number of individuals collected per plot by the 6 individuals planted. However, we could not perform this calculation for *A. elatius* due to the contamination of the seed batch. Analysis of survival rate for *L. vulgare* did not show relationships with any of the factors tested in the main data analyses.

Methods S3 *Comparison of qPCR and amplicon sequencing estimates of fungal abundance*. We designed a qPCR assay to quantify the absolute abundance of the fungal species *Paraphoma* cf. *vinacea* in the experiment. OTUs related to this species were prevalent and abundant across the experimental plots and roots of resident plants, as shown by our study and previous ones done in the same experimental plots (Mommer *et al.*, 2018; Francioli *et al.*, 2020a,b; Ampt *et al.*, 2022). Additionally, strain CBS 134643 isolated from the study site was available to us for molecular characterization (Mommer *et al.*, 2018). Although the culture had originally been identified as *Paraphoma chrysanthemicola*, we re-classified it in the closely related species *P. vinacea* by multilocus sequencing (Moslemi *et al.*, 2016). We designed species-specific primers (Pvi1F, AAGAGGGGCATTTTGGATGG; Pvi1R, TGTGCGTCATGGTGTTGATG) and a TaqMan molecular probe (PvTM1, 6FAM-TGGGGTTGTGCCGACTTTTTCGCGCCAACA-BHQ1) targeting the *tef-1α* gene sequence, which gave the highest discrimination from other *Paraphoma* species, and confirmed their specificity via PCR and qPCR assays with DNA extracted from pure cultures of CBS 134643 and strains of variable phylogenetic relatedness. We used the specific oligos to quantify *P. vinaceae* in a random subset of 98 DNA samples from roots of phytometer and resident plants, using serial dilutions of DNA from a CBS 134643 pure culture as standards for calibration. qPCRs reactions included the Premix Ex Taq™ (Perfect Real Time) qPCR kit (Takara Bio Europe SAS, Saint-Germain-en-Laye, France), 0.3 µM of each primer, 0.1 µM of the TaqMan probe, and 2 ng of DNA template in a volume of 20 µl, and were run in a CFX Connect Real-Time PCR Detection System (Bio-Rad Laboratories, Hercules, CA, USA). We calculated absolute quantities of the fungus in ng target DNA per 100 ng of total DNA by interpolation with the calibration standards.

To compare the abundances of *P. vinacea* measured by MiSeq sequencing and qPCR assays, we selected OTUs in the sequencing dataset likely to pertain to *P. vinacea* by interpolating read sequences identified as *Paraphoma* in a multilocus maximum-likelihood phylogeny containing representative sequences for all known species within the genus. Reads from OTUs that grouped with *P. vinacea* were summed and compared with the qPCR-based measurements using the Spearman's rank correlation

References cited

Ampt EA, Francioli D, van Ruijven J, Gomes SIF, Maciá-Vicente JG, Termorshuizen AJ, Bakker LM, Mommer L. **2022**. Deciphering the interactions between plant species and their main fungal root pathogens in mixed grassland communities. *Journal of Ecology* **110**: 3039–3052.

Bakker LM, Mommer L, van Ruijven J. **2018**. Can root trait diversity explain complementarity effects in a grassland biodiversity experiment? *Journal of Plant Ecology* **11**: 73–84.

Francioli D, van Rijssel SQ, van Ruijven J, Termorshuizen AJ, Cotton TEA, Dumbrell AJ, Raaijmakers JM, Weigelt A, Mommer L. **2020a**. Plant functional group drives the community structure of saprophytic fungi in a grassland biodiversity experiment. *Plant and Soil*.

Francioli D, van Ruijven J, Bakker L, Mommer L. **2020b**. Drivers of total and pathogenic soil-borne fungal communities in grassland plant species. *Fungal Ecology* **48**: 100987.

Mommer L, Cotton TEA, Raaijmakers JM, Termorshuizen AJ, Ruijven J van, Hendriks M, Rijssel SQ van, Mortel JE van de, Paauw JW van der, Schijlen EGWM, *et al.* 2018. Lost in diversity: the interactions between soil-borne fungi, biodiversity and plant productivity. *New Phytologist* **218**: 542–553.

Moslemi A, Ades PK, Groom T, Crous PW, Nicolas ME, Taylor PWJ. **2016**. *Paraphoma* crown rot of pyrethrum (*Tanacetum cinerariifolium*). *Plant Disease* **100**: 2363–2369.

van Ruijven J, Berendse F. **2003**. Positive effects of plant species diversity on productivity in the absence of legumes. *Ecology Letters* **6**: 170–175.

Weisser WW, Roscher C, Meyer ST, Ebeling A, Luo G, Allan E, Beßler H, Barnard RL, Buchmann N, Buscot F, *et al.* 2017. Biodiversity effects on ecosystem functioning in a 15-year grassland experiment: Patterns, mechanisms, and open questions. *Basic and Applied Ecology* **23**: 1–73.
