# Supplementary material for: The phylogenetic structure of plant communities drives the belowground transmission of fungal pathogens
Source: New Phytol. 2026 Apr 6;250(6):3976–89. doi: 10.1111/nph.71156 (PMC13193493; doi:10.1111/nph.71156)
Supplement: Supplementary file 2 — Fig. S5 Interactive Krona charts summarizing the taxonomic composition of fungal communities. Fig. S6 Comparison of the relative abundance of Paraphoma sp. OTUs with absolute quantification via qPCR. Fig. S7 Relationship of residents and phytometers biomass with plant richness. Methods S1 Description of the long‐term biodiversity experiment. Methods S2 Contamination of Arrhenatherum elatius phytometers with different grass species. Methods S3 Comparison of qPCR and amplicon sequencing estimates of fungal abundance. [file NPH-250-3976-s003.html]

Javascript must be enabled to view this page.

magnitude
magnitudeUnassigned

total
phytometer\_Arrhenatherum\_elatius
phytometer\_Leucanthemum\_vulgare
resident\_Achillea\_millefolium
resident\_Anthoxanthum\_odoratum
resident\_Arrhenatherum\_elatius
resident\_Briza\_media
resident\_Centaurea\_jacea
resident\_Festuca\_rubra
resident\_Galium\_mollugo
resident\_Leontodon\_hispidus
resident\_Leucanthemum\_vulgare
resident\_Phleum\_pratense
resident\_Sanguisorba\_officinalis
resident\_Trisetum\_flavescens
soil

12763346162475413952853051373409153936041915391900532966395430671231461365902457668794966427032605

12763346162475413952853051373409153936041915391900532966395430671231461365902457668794966427032605

16421642

2020

2020

2020

2020

2020

7070

7070

7070

7070

7070

15521552

15521552

15521552

15521552

15521552

1430425431331

735221

2121

2121

2121

2121

5252

5252

5252

5252

220220

220220

220220

220220

220220

113742231090

113742231090

25933226

25933226

25933226

878923864

99

99

869923855

17314

4040

5050

55

1111

2879278

2727

1592157

273273

4935444174925412996996767727315345177859179176009358136455591102442779005

3222069125216

3222069125216

4747

4747

4747

11212100

11212100

3333

791267

1632069569

1632069569

1632069569

300413310048969

300413310048969

300413310048969

300413310048969

300413310048969

4875734125625027496746730720915140176288946174849340135615531084942778502

10126864304217744624049752588421938133712

10126864304217744624049752588421938133712

668176312031778014132747265428133210

9641352

658576311621777714132747265428133158

3445101183936699175323193502

334810118393669917532396502

9797

1658445355993527864736662927168939

1658445355993527864736662927168939

1658445355993527864736662927168939

18092213683445340

1444740842313527524736662922668294

3030

6666

1072384

125125

15952198871513551

15952198871513551

15952198871513551

15952198871513551

4451133841623813994975429590715034165178112168258950129535361075534757696

4451133841623813994975429590715034165178112168258950129535361075534757696

838257838543871526157981573460812391416901242485815564929

3269044521844252512014472512466857266411762576273249

5113533863594510013783684833615154690837230723239291680

12161092886199523916961428274430812310423528324337727661123489

12161092886199523916961428274430812310423528324337727661123489

21792179

21792179

690643832881419822486576217217137887309

690643832881419822486576217217137887309

91511085439662137911449324672243717914375

417744711646211453249224201771154

2806526178927704229204137

2168112144311021303511784

108314885654714176711962439638821302627575233182060139247013014328

108314885654714176711962439638821302627575233182060139247013014328

51107821009203001022501

100527553102323

38865113699203002137

21017832

99

867214

867214

4127234732030612863313741164145214706283541052269818608235

1270104635121116911695780113

2193912191132382819990506905695202228619181730524162

168402111772128110928461242071438210330331883753

1223396275612465850442361207

4515554492166322469833432754101791532761056246136283642609

732251

2333175104343840199735

237127841454693456128248077060919843298810422878

428475691595261179

3779530252

1823243487029130392320024019625912007272853671479

2150518391309719628919324119855537587393181222921

70143422

3414016040101

3831646136412881377825524310101384

1782561167641181421

545612719393

8443474707812661613196466290228

649362343651013550191890101122186

1415515022506496276101075675319131994617604

1403515022506496276101075675319131994617484

1403515022506496276101075675319131994617484

11575135619054322658781675285114994496205

231112

2437135589641122943417121279

120120

120120

120120

13011011033117482

13011011033117482

13011011033117482

13011011033117482

66817484117482

63384549

40483722712252564167571851169736101136

40483722712252564167571851169736101136

40483722712252564167571851169736101136

40483722712252564167571851169736101136

40483722712252564167571851169736101136

929226213164677082511873563098463521751584357966923771047916150016701631963348828923065182246

1668341661

1668341661

1641341634

1641341634

1641341634

2727

2727

2727

836836

100100

100100

100100

100100

736736

463463

463463

463463

273273

273273

273273

83065127720144072497052197830082532676926

112112

112112

112112

112112

82953127720144072497052197830082532676814

170762116212516219

83121125595

83121125595

103103

103103

8587851

8587851

1010

1010

898898

682682

216216

84955817914

84955817914

11271127

11271127

3942333909

88933856

13401340

1010

868868

9393

742742

635635

635635

177177

177177

285285

140140

121121

1919

145145

145145

19345996125453317945192351322814781

8848876

88

876876

992277

482226

5151

77

77

77

77

23113673053331511351812

23113673053331511351812

16031629122414930191216319811996

16031629122414930191216319811996

66

66

152152

152152

152152

897897

875875

875875

2222

2222

44573701393539326175524952643855

44573701393539326175524952643855

44573701393539326175524952643855

625625

4949

4949

576576

486486

9090

21167399213

21167399213

21167399213

17667395713

5757

119673913

3535

3535

603642918241385103221322105225

603642918241385103221322105225

869184810829

869184810829

545810527

30822

77

28734280

516741118213593221322104396

516741118213593221322104396

315738413413593221310102473

20102748121923

44685629261225613576606612590602925231247510141176218806337822310334267

99

99

99

99

40609729163224283547605712550600925041245210115176018586337792310293932

3304822155110950265753361176752242324102366590167510866027602025247699

761858

5858

1818

219956504028

1939550408

26620

5252

1818

3434

9727965

99

2727

8667859

7070

19011179

54549

130130

66

319230205371059626365235117265139198110091651716709573907452020238990

26620

291415

99

474817021133132363762983771

37681382326194236261247235475472075

7474

3333

247112175108743228342299551376811186913479513478363376681910183104

413042311144524359517931255236217411842536044645829589

8383

895534

6161

21873402119841502275415496113235133720135

1313

88

1454612651425

1454612651425

945945

1010

935935

7344101424121772979293145275129212155057

201912171

6666101024121722979293661858212154597

55

30179222

77

1144110

2323

2727

3605050581003861319836322465115021954534431113515680

451451

451451

3553250581003861319836322465115021954534431113515162

3553250581003861319836322465115021954534431113515162

6767

6767

3458321313

3458321313

3458321313

19780122612421033287358423599361237610914916

6253716572

256256

2933716240

1111

6565

66

66

12210112

88

11410104

19027117912421033285758423599361237610914226

808864

14446

8448531

55

183131091865993285753423519311237610514003

53140369122

37153715

37153715

2323

88

36843684

157251320198174192326503737073942852814111609

27675726368616825663480156935

27675726368616825663480156935

1818

1818

1156774812510618615824710227238285281419293

1111

88

6262

2323

77611367716519

32964738537097

119559256471139

9163566109106148431066220162285281417468

1373101363

3232

1341101331

7474

7474

7474

7474

3651123639

3939

3939

3939

2556122544

1754121742

2121

1733121721

2323

2323

723723

711711

66

66

5656

99

3535

1212

10561056

10561056

10561056

96210952

96210952

945945

945945

77

77

1010

1010

135135

135135

111111

111111

2424

2424

3592898472994020191326222335600

2701738372994020191316222326769

47813254436

66

7025441

1818

34513332

1111

2828

2653325122994020191312222326327

4343431

153153

6464

8989

1414

276276

1111

2222

99

77

8888

1414

229262012124020161012222322757

77

23291732309

1358

1414

99

5454

66

66

5959

5959

5959

88526010108772

244244

77

66

231231

1168101158

1168101158

741560107345

111111

3535

14751475

1010

5768605708

1616

2525

2525

495836

495836

495836

495836

495836

253191162120611002976122857998375810494

423986596206433206489837182169

99

99

99

423086587206433206489837182169

9797

9797

386203767163

386203767163

37475658020426206489837182006

37475658020426206489837182006

35212430802332311312999

5151

5151

5151

34702430802332311312948

254516302499

254516302499

925880233231131449

1616

909864233231131449

77743

77743

5656

5656

21183

21183

5959639

23617

23617

23617

365922

365922

365922

174231941192655545284

174231941192655545284

174231941192655545284

174231941192655545284

209209

209209

3030

3030

3030

179179

179179

179179

1243029105981065422892511983

1243029105981065422892511983

674112575176540

88

88

514514

2727

2323

109109

335335

2020

1010

1010

1421125751768

1421125751768

826

826

826

107483425

99

99

98483416

824834

1616

63954

27918

27918

77

77

1212

1212

1717

1717

123123

123123

123123

29524

29524

55

2424

252632523

425425

425425

210132098

2424

7676

200131998

888342523724299198680

306222

306222

723419619675

723419619675

4867721494772

4867721494772

99

99

325423203211

325423203211

17458

945

945

88

88

159976118218220340425605395087207826020341213495318013620130268374782375178285704203

9090

9090

9090

9090

92141860

6060

6060

2424

3636

321418

1414

1414

1818

1818

19712

19712

77

77

1212

1212

1414

1414

1414

1414

123073411448413483422122147

9191

9191

9191

116961398448413482922116053

1818

1818

185185

142142

4343

253253

253253

113199375442413482922112320

991991

100835338368413482922100067

32422302

11049375210960

55

55

1075181057

10571057

1818

222662220

221762211

99

60101355992

2965291

2965291

5714135701

5714135701

1111

1111

1111

147601718168720295625598394777206525986340953491918012020130268374782375108283581572

5121433832278589241594177371152399583141382527123

113246425

113246425

5855111261317533995353

5855111261317533995353

1142157332833108768265225209

1142157332833108768265225209

59214957431

365149216

1818

77

1445139

58751

473116301632103913616278

473116301632103913616278

769455236121848

7574552361848

1212

99

99

27485124022181081710525795

5391811628476

2694612222207108117725319

105181911729429287636253

233104219

10268191172842928763217

1717

114661154682601869696136847036260100218516118542880810759462

114661154682601869696136847036260100218516118542880810759462

114661154682601869696136847036260100218516118542880810759462

157157

1818

1818

117117

117117

2222

2222

230653384982488251831069239857114041541132351012235749838431343249765999

9494

9494

2727

2727

461461

461461

173173

173173

6868

6868

141717691141217

6212619

6868

7281569114530

228413384812481351831069239857114041541132351012235749838431229249763959

227952383862473951831068139820113751541132251011335669838431229243663832

4619574113729109861127

4358427

4358427

42834

66

1010

1212

3636

283283

4646

310515271536197213242214129957

310515271536197213242214129957

310515271536197213242214129957

695676553955841632213815954488539415115384932495108835419

32816122036842148210119212554624323626650

283661108332551221034142190624273623247

36726326629857034552809

64349931619466

128128

77

408122664360

136136

2144210

581226614

27081331445251041019

27081331445251041019

33607520044173953147360595436951809465383796310527362

33607520044173953147360595436951809465383796310527362

2828

2828

188421621642485814671773518286

2323

2323

187991621642485814671773518243

187991621642485814671773518243

2020

2020

117873135926505396657929421982211717187006414103100722132872369

117873135926505396657929421982211717187006414103100722132872369

117873135926505396657929421982211717187006414103100722132872369

2643783510143282475

77

77

69861

6161

88

17969110

110110

6969

237492710143282283

1313

1020451011

1010

133152210143281249

1414

1414

1397132

125125

125125

1477

1477

99716981

99716981

99716981

916365301191749272052396

916365301191749272052396

6643649626121

2520341191723272052275

943411033310549

4949

4949

4444

4444

589710333239

589710333239

1010

1010

25134217

117117

13434100

695001394571314451878681251975348332360711774142528103552494865764241046251263

64655012261410236240

64655012261410236240

410410

410410

191539143877

191539143877

140830647337951519934186718832145681130126512441861497248102626

140830647337951519934186718832145681130126512441861497248102626

1085719020327137814283818114

1559021440

1070210020117137814243818074

16811157

16811157

2121

2121

962373

962373

43803230134102487151386805170532334133554931118428772317426931137649100339

4275632934110173415138679917045233413340493111817577231741493113764991710

9749793753815244127924

72069705

9775224832300021023526836021001411341066919817278364376714933174

9775224832300021023526836021001411341066919817278364376714933174

17940721453

17940721453

138781031724713297

138781031724713297

138781031724713297

117117

117117

117117

55

55

55

821705632715305115652189157345105615792572121316196

821515631615305115652181157345105615792572121316196

45176743522220193772

77634562491095511563218115714583613892572121312424

99

99

1028

1028

43718419

3131

3131

22518207

1818

122122

8585

181181

7474

107107

3535

3535

2020

1515

1226656

1226656

1226656

2424

2424

2424

4838125416940230774564585647433

303621978

285620178

1818

480782541634011774564585647355

90615891

471722541634011774564435646464

456843171614322

456843171614322

456843171614322

456843171614322

201708730600515402239678594197593753466237603420091234171761139766988062109121125121

3333

3333

1010

1010

2323

2323

259310432810157482081

259310432810157482081

6712669

4040

2342232

397397

2020

2020

11941043261015748684

8737932610157436

2892519245

32293

2525

2525

66

66

677677

677677

5510947967612105661144313471109414441632

17710

1010

1010

77

77

6372635

4992497

1313

431431

2424

99

22220

138138

5656

8282

1307139233110492100234770437

1307139233110492100234770437

1307139233110492100234770437

4138487343126714231409414440550

109109

109109

3492087343126714213408514434113

3491481343126714213408514434113

66

6275396263

6275396263

4545

4545

351520

351520

141141

106106

106106

1717

8989

66

66

66

2929

2929

2929

20312191

20312191

3838

3838

127127

122122

55

381226

381226

4242

1616

1616

1616

2626

1515

1515

1111

1111

4763855409379

459385409367

459385409367

459385409367

17512

17512

17512

2929

2929

2929

2929

224224

130130

130130

121121

99

5050

2626

2626

2424

1616

88

4444

4444

4444

747926949213525327481701210675435423792992241199520750158

55

55

55

225645156

4343

4343

69645

69645

6161

88

3333

2020

5252

5252

742606949199925327061701210675432123792992241199520749837

741076949199925327061701210675432123792992241199520749684

717606857198225327061664210675423023612992241199520747592

136136

221192173791181956

153153

2121

132132

149723740

1616

1616

55

55

1097237

1097237

99

99

1010

1010

1323399

1323399

1323399

88

88

88

1313

1313

1313

1092513735996

1092513735996

1212

1212

631135613

5895584

1313

99

77

1313

360360

360360

8957311

8957311

3434

3434

3434

3434

55

55

55

55

494905764120228239241172481033227354342339

48934570411122823124772481033227354341810

2121

2121

336336

336336

117117

117117

44138403

109109

33238294

212784673981165231359481011187164314536

212784673981165231359481011187164314536

149221490

147821476

1414

37348361

37348361

32725

32725

278062621102717

82626800

1954621101917

2172995666335224221469

2169295666335224221432

3737

1616

1616

2525

2525

2828

2020

88

266266

201201

6565

5566984529

5566984529

1414

3333

280658261

22944221

36145332344

236333227

236333227

236333227

1842210

1842210

8422

1010

8383

8383

8383

2424

2424

2424

2248690581415122251181305

2248690581415122251181305

2218690581415122251181275

172361071811

204669022141515781275

1212

1212

1818

1818

388942766437754947713014433815231045362646228223967

388942766437754947713014433815231045362646228223967

388942766437754947713014433815231045362646228223967

388942766437754947713014433815231045362646228223967

1696201676

1696201676

1696201676

1696201676

697222309794890941744822434616863964426934352165427192611343760552984

181112554420823532161002

3333

3333

13515120

13515120

16431105442082353216849

55840358502

107270541208233136334

1313

4349022135375242902

4349022135375242902

4129222135375240704

21982198

2137157111931947

4331491119254

379149230

1111

431924

9393

9393

1266831255

7788770

4883485

99

99

3636

3636

136136

3939

9797

164164

164164

16659183512761951966816194

20301261419191852

2541266122

23714223

153913191507

470470

470470

22036211

8383

13736128

59013577

126126

246246

21813205

2185242161

2185242161

321517

1010

22157

10983201656810757

51363504

1047020126810253

149149

1313

136136

1251381974531656812243

345345

345345

2222

2222

139014951362

139014951362

1495345944661346

1495345944661346

99

99

94855938

224224

72455714

1111

1111

488488

360360

128128

2187282571182108

2176282571182097

1111

555345549

1111

554245538

6565

6565

45969813448815876154431086753215543452

5764152528

5764152528

5422540

5422540

1213461167

10971097

874641

2929

24113228

24113228

212212

212212

5389529

4059396

6868

3636

2929

331772320957712827551526

7320647

324472320957692227551479

1818

1818

1212

77

55

221174200

84480

13717120

2323

2323

1010

1010

2323

2323

2020

2020

161310551593

161310551593

852522423774488392

852522423774488392

115115

115115

228086322162

228086322162

4466440

4466440

2574739535423614305372125447

25116391152014305372124916

4242

5894216531

277277

277277

6810215422751146659

164164

164164

4141

4141

88

88

12372822221201

524289505

6722132655

4141

525274253115139

166111111639

139171384

1414

3434

215231532068

1616

1010

66

99

99

3636

3636

47245

47245

4201292423944081282932801828112532471793336662226061751333571329034

252011734512381

50941851436

122271841193

15123

322322

71566

37520355

66

66

66

238342902998037110172112190070610116343934816683

2006157135893251671497320

21828274586223621016987188470603102343933846363

135135

9494

4141

29328516644297022074299114389417371650197611219338220430232555

119119

26118243

26326415531282702008255912748657191466176110815578213379206672

315895479272575

13534128

1212

263369979536643216474181842153765122806

1515

1515

48110471

9494

38710377

15510660122367312452651483452016812212

742810850412211565834176106394

1919

573120660626263034219225364269

233234611329536798119221530

383383

383383

55

55

62557

1111

51546

1919

1919

473473

3131

3232

4444

366366

882612860477427885

41817544102

4645536077423783

6077738892996317184251483768566491292214152080

6074338892996317184251483768566491292214152046

99

2525

203203

7878

125125

18493729261757

18493729261757

11746171511714

151717151485

1022910229

16926143881929121664416776

16736143881929121664416586

122179443412193

55

47542

1515

3535

44173841429816344301

99

99

181181

181181

3434

3434

88

2626

3819153871950152117852011437728

3819153871950152117852011437728

4949

1313

7272

184541841

1111

35019331

33273327

25258518519505251125030

131131

70952210178206883

1818

1010

1212

58125941012245255457

58125941012245255457

58125941012245255457

859025175286912611262146844445324323060399610591011038750

1414

1414

78337464732

78337464732

139224327755201094100138245323544243285910902913

139224327755201094100138245323544243285910902913

15685812248278

5252

15165812248226

695695

695695

5126506

5046498

88

509227856830975964203130

509227856830975964203130

4337426

4027395

3131

7777

66

7171

6280646815467138362517830175229979

6280646815467138362517830175229979

55

55

55

8342211801

8342211801

8342211801

2849970898628326

143143

143143

143143

2020

2020

2020

637462786550

2551317225

2551317225

24646236

24646236

99

99

1272910880

1272910880

1414

1414

1414

27685246227599

2121

2121

1818

1818

933615912

1386132

8787

70815693

114581137

77

1313

960960

77

1458137

1313

650650

639639

1111

37901443772

37901443772

369369

369369

2424

99

1515

100100

100100

10231023

10231023

11822711815

3232

1111

1140911409

2323

3477340

6161

6161

7317724

5077500

224224

67774216752

63704216345

132132

275275

221221

2626

195195

1921171904

1921171904

1921171904

55

1916171899

121121

121121

121121

121121

4329204134202

4329204134202

3634204134138

14791452

1781131352

1111

27423

69564

69564

1022416122801718525832176758154547229

9718161228017185258176758154546755

9718161228017185258176758154546755

2222

9590160728017185258176758154546632

55

9595

66

50632474

50632474

50632474

4444

2727

2727

2727

1717

1717

1717

1287570103107272201012383

1275070103107272201012258

42504465267203854

86612854

1212

2525

31063153267202735

16913156

2121

1111

4040

1616

1616

848426381075108388

842026381075108324

6464

125125

125125

125125

55

55

55

55

10229042573169095634315402506766148494166522391948233146527600518041899257354230

31359466120592161121430705

20842461851411220269

20842461851411220269

1051753559761410436

1051753559761410436

7936910714

7936910714

7936910714

790790

790790

5858

732732

14668032444131568541159219191801110655838969812041848560181041488

8487718639792684108301471952861213103264024814635167119429

24022402

276732237

78241137463631765985

731091719977988410367147195286121304024522481463516399975

568124444

3701791211258

6161

6868

199199

35313531

152152

30193019

360360

253253

253253

58019138055230770762447227259442735705895638506013918275

33413321

159159

356356

56820138055202770762447227259442735705895638506013917104

33815323

1212

557557

557557

557557

574741477422941266689701770714191419648312733789

5029297819731736115

5029297819731736115

15489664866652627358055

120120

4555608483899

10376604061552627353601

3143311

124124

253477822762342

2118227102

23237762240

3441750711349126666270176771412618421277723272

40151273418606222092

3997925268

1206251181

2853737161068126664470116171412616221277719674

1111

2493246

55

55

293225724244063319165129191858421036307337211131518631757183317183381

293225724244063319165129191858421036307337211131518631757183317183381

293225724244063319165129191858421036307337211131518631757183317183381

34346333

34346333

34346333

1915396114119040189569913857792314407466855856341865180531414163469

26998

1899

88

4213390298

28523

3933385275

1910926109918950188669913857792314407466855856341865180531414163163

216552160

110226722813

1780376078218324188669733842791814407296855856341863180531451402

211910514146

5207415561704440

8181

1744181726

25454822495

30014475998157131113080502530710748103464963184665903348032903632411499004

194194

4141

5151

9797

55

30823082

1616

30663066

26831275809155201113080222530710265103464904184615903346432903632411468145

78628758

104461038

26641975781155141113080222530710265103464904184615903346432903632411466286

6363

402402

402402

492492

434434

2020

3838

2727

1212

1515

628013134136102

3434

624613134136068

89823875

89823875

112951124

112951124

466466

429429

3737

196196

196196

55

55

92524181110169166

785785

55

1768141754

322316303

637222511106324

77

77

34460284

34460284

3188668473592582

3188668473592582

58701145855

5730115719

88

3030

102498

407421551113715143326614521296

407421551113715143326614521296

407421551113715143326614521296

407421551113715143326614521296

103994279208141037225352939841286612161791762

103884279208141037225352939841286612161791751

66

66

4040

4040

143422323658

143422323658

10159419720814103720503293981128666161791647

10159419720814103720503293981128666161791647

4040

4040

1111

1111

1111

9056569536298

714569536113

714569536113

6969

99

63656953635

1916185

1916185

1916185

340371446110532432149361924

340371446110532432149361924

314871446110532432361818

88

88

11392876

81576

32428

1515

1515

120825315017788

97925329697

1717

77

5555

15012129

17434613021042248896

17434613021042248896

501535

501535

1111

1111

255149106

255149106

255149106

47227847661483008541153382020861869516807418072146661180110105812858971151871703312620003

5656

5656

5656

5656

9285923

9085903

890890

890890

18513

18513

2020

88

88

1212

1212

361372307068733750492131269127337165229615196

21855171672662791265015337442881546

88

88

210491366721629126501533744288787

210491366721629126501533744288787

2424

2424

7743557727

7743557727

1425259317113743402134112168813645

1473144

1473144

1394259314113743402134112168813338

178311681758

113594830053743342133312168810800

602113588

55

1936187

163163

110110

4444

99

30255

30255

30255

3271835874249297294484438401135897921940210210819890

298713349424429729439512940197997921940210210818063

2626

2626

1252798

66

5151

2727

4141

59752

59752

99

99

2222

2222

1212

1212

209972870362728828736711625496594911729059510767

209972870362728828736711625496594911729059510767

29737260

260260

3737

171171

171171

8153404590972813147143010211252136677

8153404590972813147143010211252136677

174012468309379860

121411768309379341

121411768309379341

5067499

5067499

2020

2020

69762

69762

69762

1414

1414

1414

36263521273

20021179

20021179

1436380

1436380

1414

1414

55

55

66244618

66244618

62514611

37307

8585

8585

8585

8585

2104302074

632439

542430

2424

3030

99

99

204162035

204162035

158158

132861322

1818

537537

98917

98917

98917

9191

77

8763177441525898413

2121

2121

2121

8742177441525898392

8742177441525898392

8734177441525898384

88

30861236173

30861236173

30861236173

2206123685

8888

9092773872

9092773872

9092773872

9092773872

2203194402846031003181205271295672125354770132241448165146

2203194402846031003181205271295672125354770132241448165146

2203194402846031003181205271295672125354770132241448165146

1195069

221221

1542297421239

1717

55

2183894368145991003051203271295672125354770132241448163582

1313

1313

4373041717618284291114754201396185089671601737014434016451197292683568001784684722362892

177255762428215410838

2626

2626

174655762428215410812

92247762428105417

306211293

2020

25619

4040

1717

41610406

755815160356761722493393212888201053184145460861

611611

611611

749705160356761722493393212888201053184145460250

72872477105416254959553976629

12236878

6756148773496611887333912282120052794144753543

7045877114821135949273614349102712362050196774632883711282806563929

27158216935101780

27158216935101780

63034734445108035794175441588633564033947972

63034734445108035794175441588633564033947972

1949130016633

1949130016633

118511252311631106

1160112311631106

2525

3240738674234910540134955831164

3240738674234910540134955831164

6674361553145103315242752117730692383776925752158

9696

5151

6659661553145103315242752117730692383776925752011

30663331371235441832320342420671624931126260

30663331371235441832320342420671624931126260

444914431471067246620961163578422012561299620099960561999358328

176131201110786618374523385614654

68966631917010056236

340340

42001941880924646618401157477618312111287320061460451999337052

4646

58906107492161421651298772011311540321413243669

175419251710

2525

57127107302136421651298772011311540321413241934

8573616832396

2962031776

300113187

4343

150451590

6868

66

66

951580

951580

1113543109103737377

1113543109103737377

24772744714151423951

24772744714151423951

24679744714151423858

9393

278565791782257726926162489142559183735002387042652174823945472

67562

67562

272415786562254626926162488392530181834657380142651964822840444

272406786562254626926162488392530181834657380142651964822840435

99

459733111114329

459733111114329

5624444641819345692174637

7979

114114

1105105

77

531443964181934569214339

12189751428512085

77

77

12182751428512078

12182751428512078

412032526119847503746974313121760863132328105016

412032526119847503746974313121760863132328105016

412032526119847503746974313121760863132328105016

34832722045463812293084

247813018458122265

247813018458122265

1005142246329819

120143103

1867246167

2929

514121393

6363

9393

51029312517245194197478496424632416892661158846341053037010419141510

51029312517245194197478496424632416892661158846341053037010419141510

51029312517245194197478496424632416892661158846341053037010419141510

9960391855941081374529120771293225223863838329920532998180404057135712571423658

344623234283386

2004232341954

1442281432

1573635763433925761061393289323491274062

1573635763433925761061393289323491274062

7906511626174311442481519

311714

7875511626172611442481505

709709

709709

38434

38434

38985274273

38985274273

137081293162231221336421751903283764656261123619783872

543781972611191766560519037138170412016421658

827039590111245329915702835083922111163362214

177633294816936193143067640531019713124

743143

184011582337640

380145235

121121

115401447716336158141416120331879408

380839286321651483210102798

2661036697

252896697

1414

68520490190949604457515236164002118789317944830182946173633803133510148277275

6331939336185

20363368823123656

139628116331556834124491238851911416995827073910151353417269796

52132604413524889

201502228431129016575111610855710150355924318591358181159195684044

319319

32937755421674143950656157837104219985812154603334616326911764015115863

649642608941552123

106417481402327733549042493821

106417481402327733549042493821

446339551582

2632435510

905931

9337551

4848

4848

67857832662

2626

66

6465523262

2828

2828

532033

532033

73658459534435225137115849931882521352333617798572

12619107

341123

12177200048129821175925642363

66

39833801203634517790

1254107919710139

448833168442762071184396804145597224167104928

11195736480612310991758672751058922

57717871740355984789

5252

1929714172981098

2020

377021383553671

132132

132132

11811109316118181311130511635139910

11811109316118181311130511635139910

421240330110212642232

421240330110212642232

200233806206249138208210136660228183012820

24717

199993799206249138208210136660228183012803

97085228237773703499161301913493338185118391615483062

97085228237773703499161301913493338185118391615483062

800212104357735829188511918913422729827919715467384

17064178200125831112215165233920915678

153758345385375360497163348552381743633263667121470106577

646634810153136414676245578

1728115161696

26620484194

25817

1212

4412294101136146763659

2323

3402338

3402338

67372018453482142264244155

694125393229373114

6043189363165226514041

344202321666125191

331202321666125178

1313

6021336

6021336

13862932108492833444115603085055273603186106212101595541

132627162372015525756

5035401613321178234732

138138

8379813177268827624135920845329022914438230749164823

55

4513241720286518481791

43736160091960371631104825296131191467551323412

785622

1182432783433767738

1547147

1028432713433767591

312312

312312

1414

298298

184255643139481998071612166342205741124513208169787

6365194727431466130168221115762030

447487971975396130348115743464

1010

37492618339

76769

18434141752016301318139

99

114246427135251682335483618835531123413151104402

10032041753192139233525331876505112341315191015

9292

13834963332923374813295

3291868502373

3291868502373

36927099

36927099

1859528105855218

1859528105855218

158158

158158

2929

2929

91289

1919

52250

2020

6464

6464

39154337

39154337

1061987

1061987

103103

103103

103103

1128220130239748342521935129910270

1270251263

2835278

250250

2424

339339

3742372

99671993023969834252193512998962

1919

99481993023969834252193512998943

4545

4545

190765317533881492232232315996123876188558

190765317533881492232232315996123876188558

190765317533881492232232315996123876188558

165165

165165

165165

1313

1313

1313

4141

4141

4141

1045358534901812458456446200

1717

1717

69815318527

68815318517

1010

123262141156

123262141156

4558312512458456444342

4558312512458456444342

9393

9393

6363

6363

1212

1212

378049232862

378049232862

28492822662321342628

3737

3737

1072622623228

1072622623228

2705261342563

239261342252

3132311

3636

3636

2828

88

564564

564564

379379

9191

9494

79753121126137558

54211531

54211531

1661213717

1661213717

89532610

89532610

475499493349484993052262318367410345294

21243454091676

20471554091618

5151

26197

285857622544189010173829166246127409

285857622544189010173829166246127409

1452715383029532426754213898

4302344268

102251538302613242675429630

1642162

1642162

21492149

7272

20772077

55

55

55

457265611643453856

7642476345472

7642476345472

38084091143384

2805392112402

2424

3838

559442

8868878

963854179219447714648464418615521171320794879414655136521742481932129452011

4141

4141

17182151205711617018

3030

546546

16606151205711616442

6565

6565

8082423348003

44113428

7641422047575

938325179121446184648464418615471170220794879414655136521632481932123426766

1055101045

72801957256

6915686

118118

16243720111013193751786021114348092160663

1818

2222

262262

3621213600

5802163862728854223309131291356609

8131160742

703987172534417294635355783381631021220193944314512136516082481932031245745

159159

159159

543542315812002381185861969240947002090726710889

7651593272637

7651593272637

535892314311993611858619692409470020906710252

535802313411993611858619692409470020906710252

99

171221461191526

171221461191526

171221461191526

3333

3333

3333

207860213718761866

207860213718761866

4848

4848

4219419

4219419

3333

3333

195541213714761766

195541213714761766

18914175

18914175

18914175

144144

451431

45013429542114463654202521552156157742197

45013429542114463654202521552156157742197

2626

2626

394671465301034633411277552157737709

2974310945494463310867552157728125

97243776944109584

5520283121162904441564462

55

5515283121162904391564462

3838

3838

2525

2525

77

77

66

66

9999

9999

9999

9999

9999

9517173316216431135551325317465463776469412235866525

9517173316216431135551325317465463776469412235866525

9517173316216431135551325317465463776469412235866525

9517173316216431135551325317465463776469412235866525

9517173316216431135551325317465463776469412235866525

4881241763936239698120502737561872730263951184455429596

4881241763936239698120502737561872730263951184455429596

4881241763936239698120502737561872730263951184455429596

2461458292188

2321458292174

1414

4039312572804232094320121067371542537259941184453225828

4039312572804232094320121067371542537259941184453225828

7042237311077111261931186103208

7042237311077111261931186103208

40337

40337

90751311192138722195

90751311192138722195

184104170

184104170

88

88

88

88

88

2284581774823601781347983997201557104451112436346193125

2284581774823601781347983997201557104451112436346193125

2284581774823601781347983997201557104451112436346193125

4013398

4013398

17340768369511956882817265146429652190169526

17340768369511956882817265146429652190169526

3333

3333

1515

1515

1691052638

55

1641002638

31698218

31698218

8787

8787

98914011828620232241344413159253

98914011828620232241344413159253

43858169151403317731355671122771001274621713363

1006765174721192

2668805777936785311683

257995597703122576154997915609178

2736436173

1411296846231275610156373421572137

26324639176

26324639176

1818

99

99

133541331

133541331

133541331

133541331

133541331

133541331

55

55

55

55

55

55

175272137782308174607271943568711604

2018582005

2018582005

2018582005

2018582005

2018582005

15509208778230816660727194356879599

15509208778230816660727194356879599

15509208778230816660727194356879599

15509208778230816660727194356879599

15509208778230816660727194356879599

3275723844118579862423811097331212197775957148781872560310545225618

3275723844118579862423811097331212197775957148781872560310545225618

3275723844118579862423811097331212197775957148781872560310545225618

3275723844118579862423811097331212197775957148781872560310545225618

3275723844118579862423811097331212197775957148781872560310545225618

3275723844118579862423811097331212197775957148781872560310545225618

11393211115

11393211115

11393211115

11393211115

11393211115

11393211115

6673841645164933412059231811916617568320245141387714107

120120

120120

7777

7777

7777

4343

4343

2020

2323

85580

55

55

55

55

55

55

55

55

75570

75570

75570

75570

2121

2121

55

55

55

1010

1010

1010

66

66

66

1212

1212

1212

1212

1212

3030

3030

66

66

66

2424

2424

2424

36630

36630

36630

36630

36630

211211

211211

211211

211211

211211

6622341640164933412059231811916617567720245141387713603

6614641640164933412059231811916617567720245141387713526

6614641640164933412059231811916617567720245141387713526

6614641640164933412059231811916617567720245141387713526

6614641640164933412059231811916617567720245141387713526

7777

7777

7777

7777

4528482908350744214241591852330928103650108113012362438357

1010

1010

1010

1010

1010

4528382908350744214241591852330928103650108113012362438347

4528382908350744214241591852330928103650108113012362438347

4526762908350744214241591852330928103650108113012162438187

3695953250108231742194835445

124124

34884532248231742189833411

1767261051726

184184

58355

58355

369813323322415638255714631112335626

322282463322415638255714631112330959

3426243402

1327621265

1839201681651

1839201681651

1217020629711667

1217020629711667

1489515503871562442833877772852656141236146710

3838

1515

4848

1111

143143

1434635443821441852833877712852649141236141317

77

264264

286286

55

66

7878

275275

2121

125125

187187

99912987

16123138

281962813

215718174722412621014279382244705526243311168626207033

1784622119814395017749347231091917122

3030

4488224954189574823513217643895

58641983142413120214552055515

8541216728059905229245122440315458677381980

68288127946464

20794662027

110110

110110

110110

52250

52250

52250

4141

2828

2828

2828

1212

1212

77

77

99

99

1313

1313

1313

1313

1313

21072631832864083179567318130191001305689901381693843862642500702680836832251076208

167191111126146727948132793932444114707

114114

114114

114114

4747

6060

77

96647871072161074643121327148472

99

99

99

96557871072161074643121327148463

96557871072161074643121327148463

854377010727107641021327147402

11021794211051

1010

2238144141723341651850

4747

4747

4747

2191144141723341651803

37942325327

37942325327

370149347

5353

1010

307149284

23435226

23435226

1208137149316903

16610

881110121316631

3112128262

31541143121014217222960

31541143121014217222960

31541143121014217222960

31541143121014217222960

120855163755201025

120855163755201025

120855163755201025

120855163755201025

88

88

88

88

3331136286

3331136286

12693681

12093675

66

2072205

72270

135135

6262

6262

6262

6262

6262

12527677965422605717429740295083715108582

206177114725693

206177114725693

206177114725693

206177114725693

125070777953516043174294029258325108579

111437571065

111437571065

111437571065

123956774253515536174294029258325107514

58256

58256

123898774053515536174294029258325107458

123898774053515536174294029258325107458

15113138

15113138

15113138

15113138

3232

11913106

2626

2626

1212

1212

1212

1414

77

77

77

77

15661233951426

15661233951426

10363333997

10363333997

90033867

1043398

3232

482906386

56650

56650

29722

1010

77

1212

39783314

39783314

48543

48543

48543

6242350508893044371983420613801626112934541434921215843941

6242350508893044371983420613801626112934541434921215843941

6242350508893044371983420613801626112934541434921215843941

6242350508893044371983420613801626112934541434921215843941

6242350508893044371983420613801626112934541434921215843941

282323207220

282323207220

2525

2525

2525

257323207195

257323207195

257323207195

452081759481313865216603832323722342592

90628896

8181

8181

8181

82528815

5959

3131

2828

76628756

76628756

802802

452452

452452

441441

1111

350350

350350

350350

221801695440313865295238123222319741

174174

174174

174174

220061695440313865295238123222319567

90914332552224183141258305

17594637221818351610

49063872154304

18983631491863

489489

3939

396456345

374456323

2222

124301209111314402638383221810917

124301209111314402638383221810917

898747

898747

19102201719065

19102201719065

19102201719065

19102201719065

77734743

77734743

77734743

77734743

2020

2020

2020

2020

14216277201325

102071013

3635358

8484

222222

57552

7777

4949

2828

66

66

5742572

5022500

7272

265597199

1455986

1455986

1207113

2626

94787

5832035

5832035

5832035

7878

7878

7878

3006452095020993501620672441577785665868594943676270980

649564748221787695704

649564748221787695704

649564748221787695704

649564748221787695704

43581089162693643109251702862

43581089162693643109251702862

43581089162693643109251702862

43581089162693643109251702862

156432810487515509

216216

149149

149149

3232

3232

3535

3535

154272810487515293

75877248757482

275275

72257248757120

7777

1010

308643082

9754971

21112111

47541784729

4746174729

88

18247113185102718731118819032718919647341131560166195

67364130161135071425201366877

67288130161135050425201366822

125741253

461401191289504346131045758

19662112947197319582

6666

163163

762155

762155

115107130558661743111381193231641764734113154799318

10207228431010160

10207228431010160

9159212732829174271112512991401734733113154776358

337049272491105885224113119119311542923107

87786191055541310561151587733

138138

43960266419014116478115491641131040704

49051774363275214569

107107

1327332137402640242012765

26521

9528139732929339

24828340132346

123799252681551059

3535

3535

7645849169761291295433248965863382621527416966748

465005025221453816216946042

465005025221453816216946042

14615131

27318255

454693223721453816216945044

612612

6250352348113946126

6250352348113946126

309309

3333

100100

3561242338

50251048114992

2752314238

1529234116

750691921623612

709421921623598

709421921623598

412714

412714

775488474205453379

3562047120556

3562047120556

411283453323

411283453323

88

88

298318

16313

16313

1385

55

88

413413

413413

413413

7064702

6767

6767

6394635

6394635

21035471469710412713731174133631826162441612456

140173093

1717

123173076

7652131935376124127910262852624103553

7652131935376124127910262852624103553

654223

654223

1035627971782521604829628139466790

53411747131192153462742857462991

1608605573988

5722627303

7291724688

2106166186222721820

282255612639341127105212020

938105703310747

51568466395

150411135

851669

1818

11665105

10003676342121551

15220108566511322257713962

2452102554511215121328

2452102554511215121328

20119

2432102543511215121319

1276860123745712634

1198812345711921

1198812345711921

36847321

6969

29947252

412137392

2525

387137367

148195314645239926994911158911592783308778035574336176214748638224576472915520610

1158463764824093318228144582

1158463764824093318228144582

28208

28208

3692733682

3518733508

174174

3617354

3617354

1066421411409309146

1066421411409309146

64375955443111014400

64375955443111014400

2203217

20317

20317

20317

200200

200200

77

137137

5656

63256542974197408833357032102502565642821349647006107227175189147871373110225

26818250

26818250

20814194

60456

62800042569196543831737032101282514642821324946946107227175157845441200108247

28304137026381644

28304137026381644

332265612822536

332265612822536

62184841500195713831736969101282514642821324746946107227175157845441192104067

5273885796120993218443297

621151

581843368661933558298858288219195364267125934405608982717514894508107176228

346703749155165114119004681558228117489367724542

35973458651624724763131481731291

35973458651624724763131481731291

35973458651624724763131481731291

70042122495437

70042122495437

70042122495437

35138720366180186889716201210610251271720013656402722311317109923

3092002035518018688971620121058424917172001365640272231131767794

2601351026816402245451557986411540631766159939632229631764317

1818

17371741720442355118634234110331216566358

8484

416571870134446651042633554155414815014465919264

1072016763275275285932291375232584364541

17580253852902251918178196791883358514

17270534431347252957855478292256102187811514422220131725622

5415111117

5415111117

484219986161434352101981731908861221757643227

749697102616

233485020628938528139656516952327

1923327591575002185164113586702257650

5091469110759234

5901012138153213515233

5901012138153213515233

2859311328579

28561328558

28561328558

321121

321121

135541513539

1515

1515

6060

4747

1313

1347913479

1347913479

40111811

40111811

40111811

430993226406118141973751094182506

430993226406118141973751094182506

430993226406118141973751094182506

430993226406118141973751094182506

511511

511511

223223

5757

66

2424

2323

2626

1313

7474

88

88

280280

280280

1130477373320581016779734713015816080961088446

3939

3939

3939

592764168635830

592764168635830

592764168635830

16610

66

66

1010

1010

511239

421230

1414

281216

99

99

248248

9090

9090

158158

3939

3939

3535

4545

1515

1515

1515

81832520161288102

81832520161288102

39885483971

241241

46201610

322616483198

682682

163841621016077135

117117

117117

1818

1818

162491621016077

162491621016077

3434

2222

2222

1212

1212

461461

461461

461461

467362427111962171

420362427111962124

2983624271119622

122122

4747

3232

1515

8383

2424

2424

5959

5959

61980121270421677930201103361108

61980121270421677930201103361108

484021211434215279302782247733

55

135731271518321113370

19147695327812159

19147695327812159

19147695327812159

1212

1212

1212

543222

543222

543222

543222

15007287836149520352476743450988681888454848

2121

2121

2121

70052302691001333851248088688412581

66462015691001173851248088688412525

3434

65722015691001173851248088688412451

4040

3592871656

3592871656

79605493536951033913824222318842267

7770549349295103391382422174842267

328727983240948

448354969463103391382131742267

190446140

190446140

21696

21696

21696

20893196

20893196

1863183

1863183

22913

22913

2362516546

2362516546

19025165

19025165

77

77

2727

2727

1212

1212

14584197872386655

3333

3333

3333

60436043

1919

1919

441441

103103

338338

55835583

55835583

815619787238227

3333

1919

1414

102102

102102

80211978723892

793878723828

831964

332332

326326

326326

66

66

2020

2020

2020

1608152

1608152

152152

118118

2525

99

88

88

6087601

254254

178178

178178

4141

4141

3535

2626

99

204204

204204

141141

6363

4141

4141

55

3636

1376

1376

1376

1616

1616

1616

8080

8080

77

1919

5454

472221151810262273415

138138

3535

3535

1919

1919

8484

1111

7373

352211518298

23518

23518

1616

1616

31315298

1515

6161

1818

219219

423210262272979

5151

3333

1818

418110262272928

418110262272928

332751663151703023926994346241682044610916858913811293202247318188287

731954452126

5539544

5539544

17852126

5252

126126

1084265737644463643794431619286821228994112019677

5221467495

481434

3535

1106104

3297322

6811455823423917379415161710383155

6811455823423917379415161710383155

7575

4040

1717

1818

121121

121121

3939

2727

1212

35527154318852819286183994112012180

35527154318852819286183994112012180

410219389

1111

364219343

3535

347347

118118

180180

4949

737737

737737

25348328592139

25348328592139

3838

3838

55

1616

1717

51901912279177723161134022

51481912279177723161133980

1414

51341912279177723161133966

1212

1212

3030

3030

46256174602

45986174575

1010

45826174559

66

2727

2727

579610711237011334479

181184011112

181184011112

39972932633927

39972932633927

1618601091243440

5101576243392

110845101548

9099493911944147235293156088

3030

88

2222

90544939119441472352936058

55

1010

88

1361134516

32263167723682105922408

4848

77

4211328

8383

15814213614132011175

189210310227481504

7373

2020

698214673

1515

1515

537086541221365098

21813

21813

136136

125125

1111

52136541221364949

77

3838

55

48022525221364594

1212

3494016293

55

55

55

1010

1010

1010

164446144010148

164446144010148

164446144010148

826441889398221369321146274864579741

66

66

550379556168272454653

2121

548279556168272454632

771351099392667532391462741475088

588471098562662262391462741456915

17480835617386

80821787

5276521

1111

1111

4726466

5252

128128

4141

118118

3636

66

88

3030

1313

1717

2323

4444

4444

4242

4242

4242

8383

6262

88

1818

3636

2121

2121

2993661813013829761

809809

2525

99

775775

289756184828939

203203

106106

435435

1010

3838

22608622602

3232

3939

3511842327

55

30693069

102561019

1414

3535

55

6161

896896

66

3737

141126132

141126132

1111

1111

174646331730

8989

6666

2323

100100

100100

155746331541

7777

782463769

25322

112112

207207

354354

1992951897

1179561123

499499

59756541

8383

2020

2020

55

55

66139622

1717

77

3737

60039561

2222

2222

105105

1818

1818

77

1717

88

1919

1818

144144

144144

1212

8383

1111

3838

3939

3939

1515

2424

3232

3232

3232

14044151385

6868

6868

389389

1212

4242

99

3333

2424

201201

6868

268268

66

1717

6969

105105

7171

4747

3434

1313

3030

88

2222

104104

1313

9191

3131

3131

266415247

266415247

1818

1818

155155

155155

2828

2828

2222

2222

2222

734120547562

5395534

235235

3045299

174120477

174120477

2121

2121

55117250527

55117250527

55117250527

253525839415271052789

88

88

252725839415271052781

252725839415271052781

397108289

397108289

242108134

155155

1307921029707682410151312174

634826183114136246

194194

163163

77

583326183114135731

6363

8888

14381438

924924

55

2020

447447

4242

415415

3030

172172

8080

133133

159471587

1077100

197197

12061206

8484

5757

5757

32271842952458239682431

3838

1010

54643503

77

26261842952453939681873

1414

1414

1414

3131

3131

3131

8369026469429380

8369026469429380

8369026469429380

14943106

3838

3838

1114368

924349

1919

37554161160

37554161160

37554161160

545057176919710616118763496109010181632437622229714075

9985319971

1313

66

1010

1414

4646

91388

1111

147147

77

3131

771958

112112

4335428

416610694141

2828

5454

2727

2052203

3954391

369369

34628

2121

15731573

8888

1225491212

147147

393163234338

393163234338

359359

1212

347347

10097413215617381144105

10097413215617381144105

150150

150150

2871439166548732676326454710067231110402034

88

2121

45540

484133411084210150

220032011648620285419599422311542

30723

4955160312673222612540276

172172

9393

1313

55

77

16415311

107161081

1818

1010

6969

56452

3232

422422

1330103390927

391029

12273390834

1414

1212

3838

173863150248589328296461104010534562762222575050

85473319894

950113751132819399682147322569382372935

679517755622824337989373787272414201970

236582710051

729522872924

729522872924

729522872924

729522872924

729522872924
